# Elucidating the origin of decoupling in glass-forming liquids through high-temperature activation free energies

**DOI:** 10.1093/nsr/nwag366

**Published:** 2026-06-16

**Authors:** Qi-Lu Yuan, Yue-Tong Dong, Zhenyue Yang, Jack F Douglas, Francis W Starr, Zhao-Yan Sun, Wen-Sheng Xu

**Affiliations:** State Key Laboratory of Polymer Science and Technology, Changchun Institute of Applied Chemistry, Chinese Academy of Sciences, Changchun 130022, China; School of Applied Chemistry and Engineering, University of Science and Technology of China, Hefei 230026, China; State Key Laboratory of Polymer Science and Technology, Changchun Institute of Applied Chemistry, Chinese Academy of Sciences, Changchun 130022, China; School of Applied Chemistry and Engineering, University of Science and Technology of China, Hefei 230026, China; Academy for Advanced Interdisciplinary Studies, Northeast Normal University, Changchun 130024, China; Materials Science and Engineering Division, National Institute of Standards and Technology, Gaithersburg, MD 20899, USA; Department of Physics, Wesleyan University, Middletown, CT 06459, USA; State Key Laboratory of Polymer Science and Technology, Changchun Institute of Applied Chemistry, Chinese Academy of Sciences, Changchun 130022, China; School of Applied Chemistry and Engineering, University of Science and Technology of China, Hefei 230026, China; Jilin Provincial International Cooperation Key Laboratory for Polymer Processing Physics, Changchun 130022, China; MOE Key Laboratory of High Performance Polymer Materials and Technology, State Key Laboratory of Coordination Chemistry, School of Chemistry, Nanjing University, Nanjing 210023, China

**Keywords:** glass transition, dynamic heterogeneity, decoupling, activation free energy

## Abstract

It is well established that many glass-forming liquids exhibit a deviation from the Stokes–Einstein relation, which is commonly quantified by a power-law relation (so-called fractional Stokes–Einstein relation) between the mass diffusion coefficient $D$ and the momentum diffusion coefficient, corresponding to the shear viscosity $\eta$. This ubiquitous phenomenon is often attributed to dynamic heterogeneity upon cooling, characterized by the progressive growth of the average size and lifetime of dynamic clusters of particles having excessively high or low mobility. However, a predictive theoretical understanding of the non-universal power-law decoupling exponent $\zeta$ has been elusive. Here, for a wide variety of simulated glass-forming systems, we determine the power-law relation between the structural relaxation time $\tau _{\alpha }$ and the peak time $t^{*}$ of the non-Gaussian parameter, that is, $t^{*} \sim \tau _{\alpha }^{1 - \zeta }$, analogous to the power-law relation between $D$ and $\eta$ in the widely studied Kob–Andersen glass-forming liquid. We confirm the prediction from the string model of glass formation that $\zeta$ is determined by the ratio of the high-temperature activation free energies for $t^{*}$ and $\tau _{\alpha }$ in the temperature regime where dynamic heterogeneity is minimal. This finding suggests that dynamic heterogeneity is not the cause of decoupling, but rather a symptom. Our study emphasizes the role of the high-temperature liquid dynamics in understanding the fundamental mechanisms of glass formation.

## Teaser

The ‘breakdown’ of the Stokes–Einstein relation is shown to originate from the difference in the activation energies for momentum and mass transport in the high-temperature regime where dynamic heterogeneity is minimal.

## INTRODUCTION

The glass transition and glassy dynamics of liquids at low temperatures remain one of the most intriguing and challenging problems in condensed matter physics and materials science, with many unexplained phenomena that continue to puzzle the scientific community. It is widely appreciated that the dynamics of glass-forming (GF) liquids become increasingly spatially heterogeneous upon lowering the temperature ($T$) toward the glass transition temperature ($T_{\mathrm{g}}$) [1–3]. Dynamic heterogeneity refers to the occurrence of excessively ‘mobile’ or ‘immobile’ particles with mobility significantly above or below the average. Particles of excess or diminished mobility exhibit enhanced spatial correlations with particles of the same type and tend to form clusters whose size and lifetime progressively grow upon cooling [4–7]. An asymmetry in the lifetimes $t_{\mathrm{mob}}$ and $t_{\mathrm{immob}}$ of these particle clusters, which directly relate to the diffusion coefficients and relaxation times of atomic and small-molecule fluids, can be quantified by a power-law ‘decoupling’ relation, $t_{\mathrm{mob}} \sim t_{\mathrm{immob}}^{1-\zeta }$, where $\zeta$ quantifies the ‘strength’ of decoupling, with $\zeta = 0$ corresponding by definition to no decoupling [8]. An important implication of this power-law relation is that the translational mass diffusion coefficient $D$ is dominated by the mobile particles, while the shear viscosity $\eta$ is largely dominated by the immobile particles, a qualitative trend emphasized by many authors [9–12]. The expectation of coupling between these quantities arises from the non-trivial extrapolation to the atomic or molecular scale of the Stokes–Einstein prediction for the relationship of the diffusion of idealized macroscopic Brownian particles with the solvent viscosity. In atomic and small-molecule liquids, $\zeta$ quantifies the extent of the deviation from the Stokes–Einstein relation between $D$ and the structural relaxation time $\tau _\alpha$ and $\eta$ [13]. It is worth noting that in early studies, the term decoupling was applied mainly to the distinct temperature dependences of the translational and rotational diffusion coefficients in GF liquids [9,14–17], but later studies focused on the relation between $D$ and $\eta$ (and related properties), corresponding to the momentum diffusion coefficient [18], as these properties are applicable to GF liquids more broadly.

In practical terms, a large degree of decoupling means that the rate of diffusion in cooled liquids can become much greater than that expected from the Stokes–Einstein relation. Decoupling can lead to a large enhancement of the rate of homogeneous nucleation [19] and crystallization [8,20], as these processes are dominated by diffusion in cooled molecular liquids. For polymeric systems, recent studies have suggested that the decoupling relation corresponds to a power-law relation between the segmental relaxation time $\tau _{\alpha }$ and the Johari–Goldstein $\beta$-relaxation time $\tau _{\mathrm{JG}}$ [21], for which a more extended discussion is provided in Section S1. These two characteristic times thus define distinct kinetic glass transitions [22,23] for mass and momentum diffusion, respectively, serving as critical engineering parameters for material stability and processing, ranging from phase-change materials [24], battery electrolytes [25], and pharmaceuticals [26] to atmospheric science [27]. Therefore, decoupling has broad practical implications across materials science.

While it has been appreciated that the magnitude of $\zeta$ reflects an asymmetry in the persistence times of mobile and immobile particle clusters [4], the physical interpretation of $\zeta$ remains a matter of speculation. One popular idea is that $\zeta$ correlates to the fragility of glass formation [8], but many counterexamples exist; for example, ultrathin polymer films [28], knotted ring [29] and star [30] polymers, and soft spheres in variable dimensions [31] all show an inverted trend between fragility and $\zeta$. The reduction of geometrical and topological constraints on GF liquids tends to cause a decrease of fragility and the strength of decoupling. Other changes in molecular structure that enhance packing frustration alter $\zeta$ in the opposite direction. No general correlation between fragility and $\zeta$ thus exists. Decoupling evidently remains one of the great mysteries of glass formation—a puzzle within a puzzle. After considering many suggested correlations and other physically motivated hypotheses, we are forced to reconsider the physical origin of variations in $\zeta$.

In the present work, our search for the origin of decoupling is based on the general framework of the entropy theory of glass formation [32–35] and simulation results for a wide variety of GF materials, including the canonical Kob–Andersen (KA) binary mixture [36] and a range of polymeric materials, such as polymer-additive systems, star polymer melts of variable $f$ [30], and ring polymer melts of variable knot complexity [29]. These diverse systems allow us to explore a substantial range of $\zeta$ values. At the same time, the variation of $\zeta$ in these systems is important for polymer materials where $\zeta$ can change significantly due to varying nanoparticle or additive size $\sigma _{\mathrm{aa}}$, additive concentration $v$, and the interaction strength $\varepsilon _{\mathrm{ma}}$ between the polymer matrix and the additive. This accumulation of data for $\zeta$ provides a metrology for testing any model proposed for understanding the variation of $\zeta$ as a parameter for the design of new materials. Details about the simulated systems are provided in the Methods section.

## RESULTS AND DISCUSSION

Our approach to understanding $\zeta$ has its origin in the venerable Adam–Gibbs (AG) model [32] and its extension, the string model of glass formation [33], which provides a quantitative interpretation of the abstract ‘cooperatively rearranging regions’ (CRR) in the AG model. The string model has been successfully applied to simulation estimates of both $D$ and $\tau _{\alpha }$ for a wide range of fluids [35]. Our reasoning for starting from this model is simple: if the string model correctly describes these transport properties in a consistent way, then by extension it should predict the interrelation between $D$ and $\tau _{\alpha }$, and hence, $\zeta$. Strikingly, we shall see that $\zeta$ can indeed be understood to arise from differences in the high-temperature activation free energies for mass and momentum diffusion processes when there is minimal collective motion. Thus, our analysis suggests that dynamic heterogeneity observed below the onset temperature ($T_{\mathrm{A}}$) of glass formation for non-Arrhenius dynamics lacks an obvious connection to $\zeta$, contrary to our original intuition. Apart from this practical motivation for pursuing a new approach to studying decoupling, we next discuss compelling experimental observations indicating the need for a theory to address the decoupling phenomenon both above and below $T_{\mathrm{A}}$. We also have the technical problem that both $D$ and $\eta$ involve long time extrapolations in their determinations, which motivates the estimation of relaxation times related to $D$ and $\eta$ that can be determined over a large temperature range with substantially less uncertainty. We confront this technical problem first as its resolution governs how we quantify the decoupling strength in our model GF liquids.

The replacement of a linear relation between $D/T$ and $\eta$ with a power-law upon approaching the glass state is reflected in related characteristic times that can be more precisely estimated in simulations. In particular, previous studies have found that the peak time $t^{*}$ of the non-Gaussian parameter $\alpha _2(t)$ typically exhibits the same $T$ dependence as $(D/T)^{-1}$ [5], and similarly, the $\alpha$-relaxation time $\tau _\alpha$ from the self-intermediate scattering function $F_s(q,t)$ tracks $\eta$ in many systems[37,38]. Examples of the $t$ dependences of $\alpha _2(t)$ and $F_s(q,t)$ are also shown in Fig. S1a and S1b, and the $T$ dependences of $t^{*}$ and $\tau _\alpha$ are shown in Fig. S2a. Indeed, Yuan *et al.* [37] have shown the precise scaling relationship between $\eta$ and $(D/T)^{-1}$, and between $\tau _{\alpha }$ and $t^{*}$, respectively, with the prefactors precisely specified in the KA system over a wide range of $T$ under both constant density ($\rho$) and constant pressure ($P$) conditions. These results are briefly summarized in Fig. S4. Given the scaling relation between $(D/T)^{-1}$ and $\eta$, and these timescales noted above, we use $t^{*}$ and $\tau _{\alpha }$ as proxies for the time scales for mass (mobile) and momentum (immobile) diffusion, respectively [39–41].

**Figure 1.**
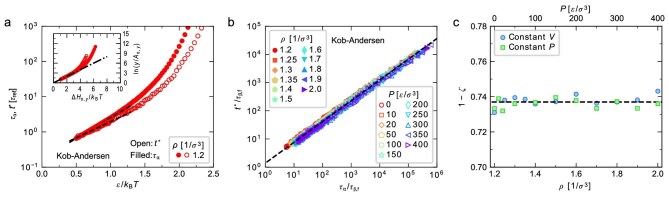
Illustration of decoupling in the KA system. (a) The Arrhenius plot of $\tau _{\alpha }$ and $t^{*}$ at $\rho = 1.2 \sigma ^{-3}$. The inset shows $\ln t^{*}$ and $\ln \tau _{\alpha }$ normalized by constants $A_{o,y}$ versus a reduced temperature defined in terms of the activation energy $\Delta H_o$ at elevated temperatures. (b) The power-law relation between $\tau _{\alpha }$ and $t^{*}$ for constant $\rho$ and constant $P$ conditions below $T_{\mathrm{A}}$. The fast $\beta$-relaxation time $\tau _{\beta , f}$ is used to normalize the time scales of each system, as discussed in Ref. [37]. (c) The $\rho$ and $P$ dependences of the decoupling exponent $1 - \zeta$. The line indicates the average of $1 - \zeta$ over different $\rho$ and $P$.

One of the important properties of $t^{*}$ and $\tau _{\alpha }$, and correspondingly, $D$ and $\eta$ of many liquids, is that the $T$ dependence of these characteristic times is generally different. Such a behavior is visually apparent in an Arrhenius plot of $t^{*}$ and $\tau _{\alpha }$. This general phenomenon is illustrated in Fig. 1a for the KA system at a representative density ($\rho = 1.2 \sigma ^{-3}$), where the activation energy, defined by the slope in the Arrhenius plot, is evidently different. We note that reported values of the decoupling exponent are normally smaller in simulations of single component atomic and small-molecule liquids that readily crystallize upon cooling than in the KA system illustrated in Fig. 1b. The particularly well-studied Lennard–Jones fluid provides an interesting example of the ‘typical’ scaling with $\zeta = 0.079$ [42], where the power-law scaling between $D$ and $\eta$ holds over a wide $T$ range extending even into the supercritical fluid regime. In this regard, it is important to recognize that decoupling is not limited to temperatures approaching the glass transition, but is in fact persistent to high temperature; that said, the differences in the $T$ dependence of transport properties are small enough at elevated temperatures that the approximation of linear coupling works rather well. Importantly, the exponent $\zeta$ tends to be much larger in liquids that readily form glasses upon cooling [13]. These broad trends would seem to suggest that frustration in intermolecular interactions that alter molecular packing enhances both the anharmonicity of these interactions and the corresponding propensity of forming a glass upon cooling, which also leads to an enhancement of decoupling. Nonetheless, these trends provide a qualitative clue about the molecular origin of this ubiquitous phenomenon. We also point out that there are multiple decoupling exponents in multi-component fluids, one for each component [43]. In the present work, we follow the convention and report the decoupling of the $A$ particles for the KA system. The assumption that the two diffusing species are not distinguished is reasonable for the KA system since the two components have similar mass diffusion coefficients. However, this situation is not always the case, and the smaller fluid components often have a much higher diffusivity and exhibit a stronger decoupling, a finding having practical significance in the relaxation of metallic glass materials [44], and presumably many other fluid mixtures. There is clearly a need for a more fundamental understanding of activation free energy parameters in the high-temperature regime where relaxation and diffusion are Arrhenius and classical transition state theory (TST) should apply.

While measurements of decoupling in the high-temperature regime are relatively scarce because relaxation times and diffusion coefficients are not extremely large, Angell and coworkers [24] have provided relevant results for chalcogenide GF materials in which the mobility is crucial to the physics of phase-change memory devices. This study indicated that the relaxation time and diffusion coefficient exhibited the Arrhenius temperature dependence with distinct activation energies over the $T$ range above the melting temperature (which is often near $T_{\mathrm{A}}$ in systems that readily crystallize [45]), along with a power-law decoupling relation in the Arrhenius regime. It is implicit from this work that decoupling at elevated temperatures derives from the different activation energies for diffusion and relaxation. A difference in the activation energies of $D$ and $\eta$ has often been observed in liquids, and this phenomenon is especially well-documented in low-mass polymers, as we discuss below.

The difference in the activation energies between diffusion and relaxation is quite understandable from a theoretical standpoint, since there is no justifiable reason for assuming the activation energies for mass and momentum transport in liquids to be identical, as essentially different molecular processes are involved in these transport processes in dense liquids [46]. The Stokes–Einstein relation lacks firm theoretical grounding in dense liquids and fails entirely in two dimensions due to the Stokes paradox [47]. While it is qualitatively plausible that relaxation requires more cooperative motion than diffusion, this intuition does not yield quantitative predictions for the activation energy difference.

We see in Fig. 1a that the difference between $t^{*}$ and $\tau _{\alpha }$ becomes amplified in magnitude below $T_{\mathrm{A}}$. This observation offers a key to understanding $\zeta$. This type of power-law scaling relation defines the ubiquitous ‘decoupling’ relation:


(1) \begin{eqnarray*}
t^{*} \sim \tau _{\alpha }^{1-\zeta },
\end{eqnarray*}

as shown in Fig. 1b. The fitted values of $1-\zeta$ are shown in Fig. 1c. $\zeta$ is found to be remarkably insensitive to the variation of $\rho$ and $P$ in the KA system, even though there are significant variations in the fragility of glass formation along different thermodynamic paths. (Figure S7 shows that this situation does not hold in charged polymer melts, however.) We show below that the variation of molecular parameters can lead to appreciable changes of $\zeta$ in other GF systems.

To better understand Eq. (1), we analyze data for $\tau _{\alpha }$ and $t^{*}$ from a wide collection of GF systems. It is evident that an explanation of the data in Fig. 1 would require a generalized TST expression in which the activation free energies for $t^{*}$ and $\tau _{\alpha }$ are distinct, but proportional, and furthermore, they must have the same $T$ dependence below $T_{\mathrm{A}}$. We now compare our simulation data for a range of GF liquids to a model of glass formation consistent with these properties that allows for a precise interpretation of $\zeta$ in terms of the activation free energies.

The string model assumes that the activation free energies $\Delta G$ for structural relaxation and segmental mass diffusion ($t^{*}$ in polymer materials) below $T_{\mathrm{A}}$ are proportional to the extent of cooperative exchange motion quantified by the average string length $L$ normalized by its value $L_{\mathrm{A}}$ at $T_{\mathrm{A}}$, leading to a $T$-dependent activation free energy, $\Delta G(T) = \Delta G_{\rm 0} z(T)$, where $z(T) = L(T) / L_{\mathrm{A}}$. Freed [34] has offered the derivation of a generalized TST that accommodates collective atomic motion consistent with the string model. Hence, $\tau _{\alpha }$ and $t^{*}$ should obey the generalized Arrhenius relations:


(2) \begin{eqnarray*}
\tau _{\alpha } = \tau _{\rm 0} \exp \left[\frac{\Delta G_{{\rm 0},\tau _{\alpha }}}{k_{\mathrm{B}}T} z(T) \right]
\end{eqnarray*}

and


(3) \begin{eqnarray*}
t^{*} = t^{*}_{\rm 0} \exp \left[\frac{\Delta G_{{\rm 0},t^{*}}}{k_{\mathrm{B}}T} z(T) \right],
\end{eqnarray*}

where $\Delta G_{{\rm 0},\tau _{\alpha }}$ and $\Delta G_{{\rm 0},t^{*}}$ correspond to the high-temperature ($T \ge T_{\mathrm{A}}$) activation free energies for $\tau _\alpha$ and $t^{*}$. The prefactors $\tau _{\rm 0}$ and $t^{*}_{\rm 0}$ can be determined directly from the simulation data of $\tau _\alpha$ and $t^{*}$ at $T_{\mathrm{A}}$. We again emphasize that the activation energies for mass and momentum diffusion are normally observed to be different in the Arrhenius regime. This situation not only applies to atomic GF liquids such as the KA system, but has also been frequently noted in the well-studied case of relatively low molecular mass polymer liquids such as alkane melts [48]. This finding is inconsistent with the Stokes–Einstein relation in the high $T$ Arrhenius regime, where the effect is often small enough so that it can reasonably be neglected. Note that the limited $T$ range available to obtain the activation energies in Fig. 1a can be a source of uncertainty in the estimation of these energetic parameters.

The determination of the extent $L$ of string-like collective motion and the constraints in various GF liquids have been discussed previously [33], which we also briefly summarize in Section S5. An illustrative example for the $t$ dependence of the number-averaged string length $\langle s(t) \rangle$ is shown in Fig. S1c, and an example for the $T$ dependence of $L$ is shown in Fig. S2b.

**Figure 2.**
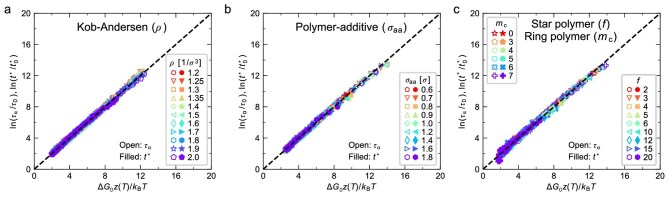
Test of the string model of glass formation for the structural relaxation time $\tau _{\alpha }$ and the peak time $t^{*}$ of the non-Gaussian parameter in various GF liquids. The results for (a) the KA system with varying density $\rho$, (b) a polymer-additive system with varying additive size $\sigma _{\mathrm{aa}}$, and (c) a star polymer melt with varying arm number $f$ and a knotted ring polymer melt with varying minimal crossing number $m_c$. $y$ refers to either $\tau _{\alpha }$ or $t^{*}$. Lines indicate the string model relation in Eqs (2) and (3).

Before making use of the string model explicitly, it is important to first validate its applicability to our data. This requires us to test the ability of the string model to describe the $T$ dependence of $t^{*}$, which has not been previously considered, as well as the consistency of this $T$ dependence with the string model prediction for the variation of $\tau _{\alpha }$ with this measure of collective motion. Figure 2 shows the string model description of $\tau _{\alpha }$ and $t^{*}$ for selected systems. In all cases, we find agreement with the predicted relation between relaxation times ($\tau _{\alpha }$ and $t^{*}$) and $L$. In the present work, we adopt the simplifying assumption that the high-temperature activation free energies for $\tau _{\alpha }$ and $t^{*}$ can be treated as approximately constant, an assumption adopted in the original AG model [32]. In Fig. S5, we show that this approximation does not significantly degrade the fitting of the string model to our simulation data.

**Figure 3.**
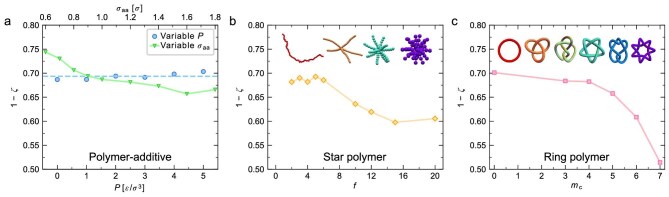
Estimate of the decoupling exponent for various systems. The results for (a) a polymer-additive system with varying $P$ and $\sigma _{\mathrm{aa}}$, (b) a star polymer melt with varying $f$, and (c) a knotted ring polymer melt with varying $m_c$. The insets in panels (b) and (c) show schematic images of the polymers.

We next examine the variability of $\zeta$ in different GF liquids. As noted above, $\zeta$ is highly insensitive to variations of both $\rho$ and $P$ in the KA system, a feature that may reflect a deeper aspect of the model, but is unhelpful at present for exposing the physical origin of $\zeta$. We therefore turn to other GF systems. A representative example is a flexible polymer matrix (*m*) with molecular additives (*a*) of variable size, where $\zeta$ depends on the size $\sigma _{\mathrm{aa}}$ of the additives. Our simulations of a composite material are primarily limited to a fixed representative concentration $v = 0.1$ and a fixed interaction strength between the polymer matrix and additives, $\varepsilon _{\mathrm{ma}} = 1.5 \varepsilon _{\mathrm{aa}} = 1.5 \varepsilon _{\mathrm{mm}}$, where $\varepsilon _{\mathrm{aa}}$ and $\varepsilon _{\mathrm{mm}}$ correspond to the additive–additive and polymer–polymer interaction strengths, respectively. We see in Fig. 3a that varying $P$ for a given additive size has little effect on $\zeta$, but varying the additive size at fixed $P$ allows $\zeta$ to change over a considerable range, $0.65 \lesssim 1 - \zeta \lesssim 0.75$. The effect of varying the additive concentration is briefly considered in Fig. S7c, where the polymer–polymer and polymer–additive interactions are the same as in Fig. 3a. Varying the additive concentration can also greatly influence $\zeta$. We expect this variation to be even larger in semi-flexible polymer melts when the polymer–additive interaction strength is varied over a wide range. The parameter space involved in polymers with additives is evidently large.

Changing molecular topology while keeping the monomer structure invariant can also appreciably alter $\zeta$. Figure 3b and c shows significant variations in $\zeta$ in many-arm star and knotted ring polymer melts. We suggest that this variation is related to the appreciable changes in molecular packing, as observed in previous studies [29,30]. Notably, the increase in topological complexity as quantified by $f$ or $m_c$ leads to an increase in $T_{\mathrm{g}}$, but to a decrease in fragility, so that the extent of decoupling can be anti-correlated with fragility [29,30].

Having demonstrated the power-law decoupling and the validity of the string model to describe $\tau _{\alpha }$ and $t^{*}$ for all the systems, we consider how these observations are connected. It is readily evident that Eqs. (2) and (3), derived from the string model, are consistent with decoupling in the form of Eq. (1). More specifically, Eqs. (2) and (3) mathematically imply


**Figure 4.**
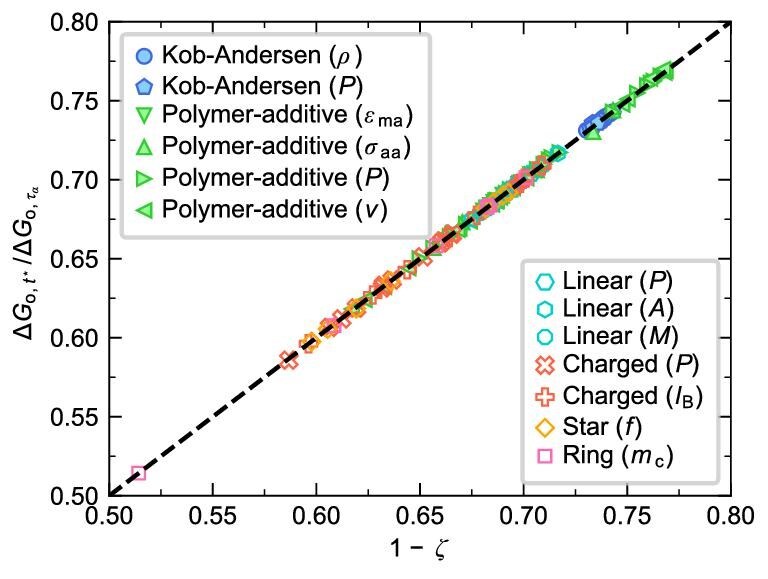
Relation between the decoupling exponent $1 - \zeta$ and the ratio of the high-temperature activation free energies, $\Delta G_{{\rm 0}, t^{*}} / \Delta G_{{\rm 0}, \tau _{\alpha }}$, determined from the string model of glass formation. The analysis considers the KA system with varying $\rho$ and $P$, a polymer-additive system with varying $\varepsilon _{\mathrm{ma}}$, $\sigma _{\mathrm{aa}}$, $P$, and $v$, a linear polymer melt with varying $P$, chain rigidity $A$, and molecular mass $M$, a charged polymer melt with varying $P$ and Bjerrum length $l_{\mathrm{B}}$, a star polymer melt with varying $f$, and a knotted ring polymer melt with varying $m_c$. The line indicates the direct equivalence between the two quantities.

(4) \begin{eqnarray*}
1 - \zeta = \frac{\Delta G_{{\rm 0},t^{*}}}{\Delta G_{{\rm 0},\tau _{\alpha }}} + \mathcal {\rm 0}\left(\ln \frac{\tau _{\rm 0}}{t_{\rm 0}^{*}}\right),
\end{eqnarray*}

where the correction term can be neglected since $\tau _{\rm 0}/t_{\rm 0}^{*} \approx 1$. In physical terms, this equation means that the decoupling exponent below $T_{\mathrm{A}}$ is determined by the ratio of the activation free energies $\Delta G_{{\rm 0},\tau _{\alpha }}$ and $\Delta G_{{\rm 0},t^{*}}$, whose values are observed to be different even in the Arrhenius regime. This result is a non-trivial prediction of the string model that has not been well-appreciated previously that offers a novel test of this model of glass formation. That said, an earlier work on ultra-thin polymer films [49] recognized the importance of the high-temperature activation barrier in determining mobility gradients approaching the glass transition, and Refs. [8,50] also noted the importance of activated transport for understanding the decoupling phenomenon. Figure 4 shows that all our data are remarkably well-described by Eq. (4). Our analysis thus demonstrates that, to a leading order, decoupling can be attributed to the disparity in activation barriers for structural relaxation and translational diffusion that exist in the $T$ regime above $T_{\mathrm{A}}$, where little dynamical heterogeneity exists. While the difference in activation energies for $t^{*}$ and $\tau _{\alpha }$ exists even in the Arrhenius regime above $T_{\mathrm{A}}$, fitting our data using the string model allows us to obtain more precise estimates of the activation energies in the high $T$ regime above $T_{\mathrm{A}}$ because of the much larger $T$ range in fitting and much larger variations of $t^{*}$ and $\tau _{\alpha }$ below $T_{\mathrm{A}}$ where collective motion amplifies the magnitude of the activation energies.

Our results on $\zeta$ are qualitatively in line with the trend indicated by Ngai’s Coupling Model [51], where the extent of decoupling is the defining relationship. In this conceptual model of the dynamics of condensed materials with many-body interactions, the more ‘constrained’ the dynamics of a material system are, the larger $\zeta$ becomes. While the Coupling Model offers no theoretical means of calculating $\zeta$, the phenomenological success of this scaling argument explains its continued interest and discussion [52]. Based on this type of argument and observed experimental correlations, Ngai and coworkers [53] have argued that $\zeta$ is not directly related to dynamic heterogeneity, the most common rationalization of decoupling and a viewpoint that we also emphasized in the past [30]. It was a combination of these arguments, along with our failure to find a universal correlative relationship between $\zeta$, fragility or other aspects of glassy dynamics noted previously, that finally prompted us to consider a different perspective to understand the physical origin of the variation of $\zeta$ in GF liquids.

Our finding is thus consistent with the arguments of Ngai *et al.* [53] that $\zeta$ is not directly derived from dynamic heterogeneity. On the other hand, the anisotropies in the magnitudes of the activation energies for $\tau _{\alpha }$ and $t^{*}$ are not inconsistent with the appearance of dynamical heterogeneity for $T< T_{\mathrm{A}}$, where the disparity in activation energies becomes greatly enhanced by the growing scale of collective motion. It is notable that the deviation from the Stokes–Einstein relation is frequently observed in phase-change GF materials over a $T$ range appreciably above $T_{\mathrm{A}}$ [54], an observation which, by itself, makes any general causal relationship between decoupling and dynamic heterogeneity dubious.

The observation in Fig. 4 of a general relation between $\zeta$ and the activation parameters shifts the problem of understanding decoupling to understanding the relative magnitudes of the activation free energies for diffusion and relaxation in the high $T$ Arrhenius regime. In this regime, particle motion in the liquid is known to involve collective motion in the form of diffusing vortices that are responsible for the long-time power-law decay of the velocity autocorrelation function [46,55,56] and stress autocorrelation function from which $D$ and $\eta$ are obtained, so it is understandable that some decoupling persists in the fluid at high $T$ [24,48,54], even if it is less conspicuous in experimental measurements. Notably, the computational observations of Alder and coworkers [46] revealed that correlated particle motion exists in fluids even in the high $T$ regime where Brownian motion was formerly thought to apply, and this phenomenon remains under investigation [57]. Collective particle motion is evidently inherent to the liquid state of matter, but the extent of this effect and the associated long-time tail phenomenon become greatly amplified in cooled liquids where dynamic heterogeneity becomes appreciable [58]. Nonetheless, these highly simplified models that completely neglect collective motion, such as Brownian motion theory, provide useful approximate models of the dynamics of liquids above $T_{\mathrm{A}}$ where the effects of thermal fluctuations, symptomatic as dynamic heterogeneity, can be reasonably neglected.

We emphasize that the key to the common $T$ dependence of the activation free energies for $\tau _{\alpha }$ and $t^{*}$ in the string model is derived from their common dependence on the fluid configurational entropy $S_c$, which would also provide a suitable perspective for understanding the physical origin of Eq. (1). Unfortunately, the numerical estimation of $S_c$ is challenging computationally, and there remains some debate on the proper approach for evaluating $S_c$. In particular, the point of contention involves the treatment of the effect of anharmonicity of the interparticle interaction in the estimation of the vibrational contribution to the fluid entropy required, a technical matter discussed by Das and Sastry [5]. In contrast, $L$ can be readily quantified for GF liquids using simulations, which is the main reason that we focus on the string model.

Our interpretation of $\zeta$ relies on the string model prediction that the $T$ dependence of the activation free energy is directly related to the $T$ dependence of $L$. To validate this assumption, it is helpful to directly compare the $T$ dependence of the activation free energy of the structural relaxation time to that of the independently estimated string length. As expected from classical TST [59], which treats structural relaxation as a thermally activated process, the activation free energy $\Delta G(T)$ can be defined formally as


**Figure 5.**
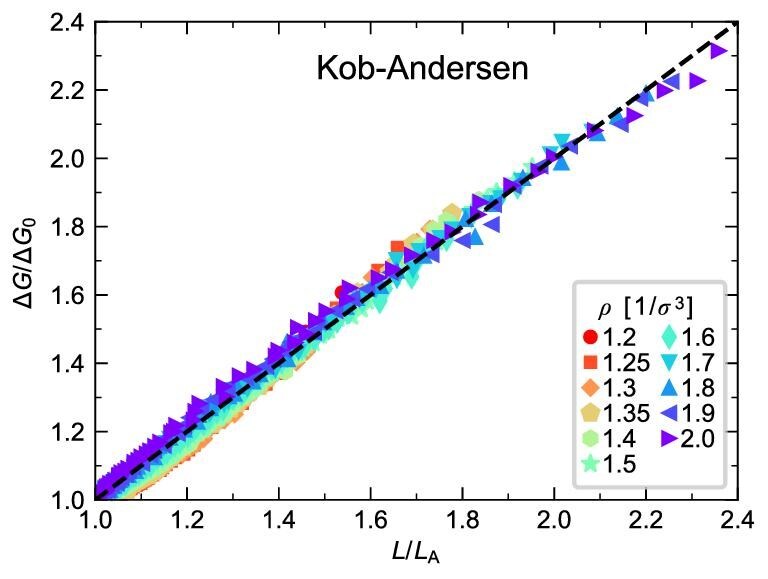
Alternative test of the string model. The plot shows a parameter-free comparison between the temperature dependences of the normalized activation free energy $\Delta G / \Delta G_0$ and the normalized average string length $L/L_{\mathrm{A}}$ below $T_{\mathrm{A}}$ for the KA system at varying densities $\rho$.

(5) \begin{eqnarray*}
\Delta G(T) \equiv k_{\mathrm{B}} T \ln (\tau _{\alpha } /\tau _0),
\end{eqnarray*}

where $\tau _0$ represents the vibrational relaxation time, typically taken to be $\tau _0 \approx 10^{-13}$ s for small-molecule fluids. Based on TST, AG [32] further hypothesized the existence of abstract CRR in GF liquids. The size $z$ of CRR was assumed to scale proportionally with the activation free energy via the relation, $z = \Delta G/\Delta G_0$, where $\Delta G_0$ represents the activation free energy in the high $T$ Arrhenius regime. Figure 5 shows a direct comparison between $\Delta G/\Delta G_0$ and $L/L_{\mathrm{A}}$ below $T_{\mathrm{A}}$ in the KA system under a wide range of constant density conditions, with the prefactor being $\tau _0 = 0.12 \tau$. This comparison between independently determined quantities validates the string model in another fashion. Such a relationship was validated previously for a coarse-grained linear polymer melt [33]. We also note that the string length has been found to be invariant in its average length in thin films, consistent with this quantity being related to the configurational entropy, a long wavelength thermodynamic property [60]. Notably, the vibrational relaxation time is on the order of magnitude $\tau _0 \sim 10^{-13}$ s, which accords with the assumed order of magnitude in both the generalized entropy theory [35], and Eyring’s TST [59] estimated of this quantity.

As a final point, the role of immobile particle clusters (which persist on the timescale of the structural relaxation time) in decoupling has not been widely explored [4]. Most studies of dynamic heterogeneity have focused on the identification and characterization of mobile particle clusters and string-like collective exchange motion. The mobile particle clusters persist on a timescale on the order of the diffusion time $t^{*}$, and simulations to longer times are required to study the immobile particle clusters, a fact that has no doubt contributed to their more limited study. This complementary type of dynamic heterogeneity deserves more attention in connection to decoupling, as it has been argued that immobile particle clusters can give rise to decoupling in the form of the fractional Stokes–Einstein relation in GF liquids [13]. More specifically, decoupling can be derived from hydrodynamic theory, where the presence of a finite concentration of immobile particles (such as hard spheres) in a liquid of relatively small molecules leads inherently to an asymmetry in how the mass and momentum diffusion coefficients are influenced by the presence of such particle ‘heterogeneities’. While this simple model is instructive about the general physical origin of decoupling from a general hydrodynamic perspective, the model is oversimplified in that it neglects the finite lifetime of the immobile clusters modeled by simple spherical particles of a larger size and their polydispersity in size and shape. We have thus not abandoned the idea that $\zeta$ can be related in some fashion to dynamic heterogeneity. Along similar lines, since the four-point correlation function $\chi _4$ is dominated by immobile particle clusters [4], we suggest that some relation might exist between the peak height of $\chi _4$, often interpreted heuristically as being related to the average mass of the immobile particles, and $\zeta$. It seems likely that these immobile clusters give their own distinct contribution to the dynamics of GF liquids. A recent dielectric relaxation study has indicated that the ratio of the activation free energies of $\tau _{\alpha }$ and $\tau _{\mathrm{JG}}$ for polymeric GF liquids correlates strongly with the magnitude of $\chi _4$ [61], which is significant given the strong correlative relationship that exists between $\tau _{\mathrm{JG}}$ and $t^{*}$ (see Section S1).

## CONCLUSIONS

We have systematically examined the power-law relation between the lifetimes of ‘mobile’ and ‘immobile’ particle clusters in a wide variety of GF systems. We found that the power-law decoupling relation, $t^{*} \sim \tau _{\alpha }^{1 -\zeta }$, holds for all systems studied. In some cases, $\zeta$ is remarkably insensitive to thermodynamic conditions, but in other systems, $\zeta$ is considerably sensitive to interactions with the additives and molecular topology. We utilized the string model of glass formation to gain insight into the variation of $\zeta$, since this model makes precise predictions for the interrelation between $t^{*}$ and $\tau _{\alpha }$ in the temperature regime below $T_{\mathrm{A}}$ where relaxation is non-Arrhenius. Specifically, we found that $\zeta$ could be understood in terms of the ratio of the activation free energies for mass and momentum diffusion. While this new correlation does not provide a simple physical explanation of $\zeta$ in terms of molecular parameters, it does provide a relatively tractable approach for estimating $\zeta$. Our study points to the fact that the high-temperature liquid dynamics is crucial for understanding the fundamental mechanisms of glass formation. Such a topic would be ideal for machine learning methods as high throughput simulations are feasible because of the relatively short relaxation timescales under such conditions.

## METHODS

### System details

All simulated systems are modeled using a truncated-and-shifted Lennard–Jones potential for non-bonded interactions. For polymeric systems, adjacent beads are connected via the finitely extensible nonlinear elastic potential, with chain rigidity controlled by an additional bending potential. In systems containing ions, charged beads interact through a long-range Coulomb potential. We investigate a diverse range of systems: the KA binary mixture, linear polymer melts of varying length and rigidity, star polymers, knotted ring polymers, charged polymer melts with counterions, and polymer-additive composites. Specific details for each system are provided in Section S2.

### Simulation protocol

Molecular dynamics simulations were performed in three dimensions under periodic boundary conditions using the LAMMPS package [62,63]. Temperature and pressure were regulated by Nosé–Hoover barostat and thermostat, respectively. Following equilibration at a high-temperature, systems were quenched at a constant rate to investigate glass formation. Additional details are given in Section S2.

## Supplementary Material

nwag366_Supplemental_File
