# Supplementary material for: Elucidating the origin of decoupling in glass-forming liquids through high-temperature activation free energies
Source: Natl Sci Rev. 2026 Jun 16;13(15):nwag366. doi: 10.1093/nsr/nwag366 (PMC13471036; doi:10.1093/nsr/nwag366)
Supplement: Supplementary file 1 [file nwag366_supplemental_file.pdf]

Supplementary Material for "Elucidating the origin of decoupling in glass-forming liquids through high temperature activation free energies"

Qi-Lu Yuan,[1,2] Yue-Tong Dong,[1,2] Zhenyue Yang,[3] Jack F. Douglas,[4,*]
Francis W. Starr,[5,†] Zhao-Yan Sun,[1,2,6,‡] and Wen-Sheng Xu[7,§]

[1] *State Key Laboratory of Polymer Science and Technology, Changchun Institute of Applied Chemistry, Chinese Academy of Sciences, Changchun 130022, China*

[2] *School of Applied Chemistry and Engineering, University of Science and Technology of China, Hefei 230026, China*

[3] *Academy for Advanced Interdisciplinary Studies, Northeast Normal University, Changchun 130024, China*

[4] *Materials Science and Engineering Division, National Institute of Standards and Technology, Gaithersburg MD 20899, USA*

[5] *Department of Physics, Wesleyan University, Middletown CT 06459, USA*

[6] *Jilin Provincial International Cooperation Key Laboratory for Polymer Processing Physics, Changchun 130022, China*

[7] *MOE Key Laboratory of High Performance Polymer Materials and Technology, State Key Laboratory of Coordination Chemistry, School of Chemistry, Nanjing University, Nanjing 210023, China*

[*] jack.douglas@nist.gov; NIST Fellow, emeritus.

[†] fstarr@wesleyan.edu

[‡] zysun@ciac.ac.cn

[§] wsxu@nju.edu.cn

S1. JOHARI–GOLDSTEIN RELAXATION AND NON-GAUSSIAN PARAMETER IN POLYMERIC LIQUIDS

The phenomenon of decoupling is more complicated in polymeric GF liquids than in atomic and molecular GF liquids because the segmental relaxation time can differ significantly from relaxation on the spatial scale of the polymer as a whole, an effect that leads to a breakdown of the time-temperature superposition when the difference between these relaxation times becomes large [1–4]. In particular, the segmental dynamics of polymers exhibit a highly variable fragility with polymer structure and chain rigidity that is often much larger than that of molecular liquids, while the fragility of the overall chain dynamics related to the overall rate of polymer diffusion and the polymer melt viscosity shows relatively little variation [4–8]. As a consequence, the extent of decoupling between diffusion and viscosity found even in a characteristic fragile polymer material such as unentangled polystyrene has been found to be rather weak [4, 9]. On the other hand, there is substantial evidence that decoupling still exists in connection with the polymer segmental dynamics. As discussed in the main text, this type of power-law scaling relation generally exists between τ_α and t^*, which relates to the dynamics of segmental motion rather than the diffusion of the chain as a whole. The power-law relation between τ_α and t^* is much like that found in atomic and molecular fluids, where this relation is directly related to the fractional Stokes-Einstein relation, as discussed in depth for the Kob-Andersen system [10]. This situation raises the question of the measurable consequences of such a diffusive segmental process occurring on a much shorter timescale than the segmental relaxation time and on the still much larger timescale for the chain to diffuse a distance on the order of the chain radius of gyration. We next discuss evidence suggesting the existence of such a timescale.

Recent studies have hypothesized that the Johari–Goldstein β-relaxation time $\tau_{\rm JG}$ [11–13], a key relaxation process observed generally in GF liquids whose physical interpretation has been much disputed, corresponds to the peak time t^* of the non-Gaussian parameter $\alpha_2(t)$ [14, 15], which, in turn, has been found to correlate strongly with the lifetime of mobile particle clusters in cooled molecular liquids and polymers [16, 17]. We previously reviewed evidence supporting this correspondence between t^* and $\tau_{\rm JG}$ [18, 19], noting that t^* scales universally with a power of τ_α in uncharged polymers over a wide range of T and P. A similar "universal scaling" between $\tau_{\rm JG}$ and τ_α has been observed in many measurements of GF

liquids in which P is varied over a large range [20]. While these observations are suggestive, they fall short of definitively establishing the precise correspondence between t^* and τ_{JG}. We nonetheless think that the evidence is sufficiently strong to adopt this correspondence as a working hypothesis. Note that our interpretation of this "precursor process" to segmental relaxation does not require the invocation of any new exotic relaxation process specifically related to glass formation, but only the recognition that mass and momentum transport processes of segmental relaxation occur on timescales distinct from the molecule as a whole or associated complex of molecules. There is thus no "mystery" about the physical origin of the Johari–Goldstein relaxation process from a hydrodynamic perspective. It is also evident why this relaxation process becomes prevalent and dominates below the glass transition temperature T_{g}, because segmental relaxation is largely attenuated due to the large value of the structural relaxation time in materials in their glass state.

The parallel between these characteristic relaxation times of glass formation is further supported by the scaling relation $\tau_{\mathrm{JG}} \sim \tau_\alpha^{1-\zeta}$ with $1-\zeta \approx 0.71$ in a coarse-grained polymer melt [20], which is notably consistent with our finding of the decoupling exponent between t^* and τ_α in pure polymer melts [19]. Somewhat smaller values, $1-\zeta = 0.69 \pm 0.01$ or 0.68 ± 0.01, were also reported in Ref. 20, depending on the method used to estimate the relaxation times. This exponent estimate is also consistent within numerical uncertainty with the estimated decoupling exponent ζ between t^* and τ_α for the linear polymer melt in our previous work [19], further reinforcing the hypothesized quantitative link between τ_{JG} and t^*. We also note that the power-law scaling relation between τ_α and τ_{JG} is essentially invariant to the variation of pressure and that these characteristic times exhibit thermodynamic scaling [21] to a good approximation in diverse GF liquids [22, 23]. We find that this scaling property is also shared by t^* in a range of simulated GF liquids [10, 19, 24]. The circumstantial evidence relating t^* and τ_{JG} is thus rather strong.

The proposed relation between τ_{JG} and t^* also offers insight into the broadly recognized significance of τ_{JG} in diverse engineering applications of polymer materials. The Johari–Goldstein β-relaxation supersedes in significance the primary α-relaxation below T_{g} by the simple fact that molecular diffusion remains an active channel for material relaxation, while stress relaxation is largely shut down because of the large magnitude of τ_α below T_{g}. At even lower temperatures where τ_{JG} and presumably t^* become on the order of 100 s, we may expect this default channel of relaxation by diffusive particle displacement to

shut down as well, leading to material embrittlement [25] and other significant changes in dynamic processes, such as chemical reactivity reliant on molecular diffusion. In polymer materials, the diffusive particle motion in polymer at a segmental level contributing to the Johari–Goldstein relaxation process can be expected to involve a combination of particle translational and rotational motions [26]. The characteristic times t^* and τ_α can be used to define *two* glass transition temperatures [27–30] at which mass and momentum diffusion processes, respectively, become sluggish, and below which non-equilibrium effects such as physical aging phenomena become prevalent. These dual effective glass transitions, defined by somewhat arbitrary kinetic conditions, are evidently engineering parameters rather than temperatures having intrinsic fundamental thermodynamic significance, but this situation does not detract from the practical significance of these parameters in applications.

We note that one objection to the correspondence between τ_{JG} and t^* is that τ_{JG} is often observed to have an approximately Arrhenius temperature dependence [31–37] rather than the strongly non-Arrhenius temperature dependence often reported for both τ_α and t^*. However, this difference can be understood from the difference in the temperature regimes in which these relaxation times are normally estimated [32]. Most reports of τ_α are made above T_{g} where equilibrium measurements can be readily performed, while most measurements on τ_{JG} are made below T_{g} where this relaxation process becomes prevalent because of the very large value of τ_α in this temperature regime as noted above. The string model of glass formation predicts a second approximately Arrhenius regime at low temperatures below T_{g} [38]. This low temperature Arrhenius regime is also predicted from the generalized entropy theory (GET) for ideally flexible chains [39]. (The GET model of semi-flexible polymers leads to an apparently spurious vanishing of the configurational entropy S_c at a finite temperature, and a corresponding divergence of the structural relaxation time due to the perturbative treatment of chain stiffness in the lattice polymer model [39].) There have been many reports of an approximately Arrhenius temperature dependence of τ_α at temperatures near and below T_{g} [31]; e.g., McKenna and coworkers have recently reported deviations from non-Arrhenius dynamics deep in the glass state [40, 41]. However, the interpretation of these observations is not yet clear. The Johari–Goldstein relaxation process has been observed to transition to a non-Arrhenius temperature dependence above T_{g}, so there is no real contradiction between the temperature dependences of τ_{JG} and t^* [23]. We may then understand why τ_{JG} and τ_α are often reported to have a power-law relation regardless

of the temperature range investigated, as found in simulation estimates of t^* and τ_α over a temperature range far above T_g. Importantly, this mass and momentum relaxation time correspondence and the existence of a low temperature Arrhenius relaxation regime together imply that that the ratio of these activation energy ratios in the high and low temperature regimes should be the equal. This is practically important in the case of polymers since polymer materials tend to become thermally unstable for temperatures as high as T_A[42]. Finally, we note that the correspondence between t^* and $\tau_{\rm JG}$ naturally explains why strings have been observed to be associated with the Johari–Goldstein relaxation [14, 15, 43, 44], i.e., the relaxation processes are possibly *identical.*

S2. SYSTEM AND SIMULATION DETAILS

S2.1. Interaction potentials

All the systems that we simulate employ a truncated-and-shifted Lennard-Jones (LJ) potential between nonbonded particles given by,

$$U_{\alpha\beta}(r) = 4\varepsilon_{\alpha\beta}\left[\left(\frac{\sigma_{\alpha\beta}}{r}\right)^{12} - \left(\frac{\sigma_{\alpha\beta}}{r}\right)^{6}\right] + C(r_{\rm c}), \tag{S1}$$

for $r < r_{\rm c}$ and zero otherwise, where r is the particle separation, the indices α and β represent particle type, and $\sigma_{\alpha\beta}$ and $\varepsilon_{\alpha\beta}$ set the length and energy scales, respectively. The constant $C(r_{\rm c})$ ensures that $U_{\alpha\beta}(r)$ changes to zero smoothly at the cutoff distance $r_{\rm c}$. We choose $r_{\rm c} = 2.5\sigma_{\alpha\beta}$ to include attractive nonbonded interactions. For systems having only one particle species (e.g., linear, star, and ring polymer melts), we utilize the following notations, $\varepsilon = \varepsilon_{\alpha\alpha}$ and $\sigma = \sigma_{\alpha\alpha}$.

Except for the KA system, there are also bonded interactions between adjacent beads along polymer chains that are governed by the finitely extensible nonlinear elastic (FENE) potential [45, 46],

$$U_{\rm FENE}(r) = -\frac{1}{2}k_b R_0^2 \ln\left[1 - \left(\frac{r}{R_0}\right)^2\right] + 4\varepsilon_{\rm mm}\left[\left(\frac{\sigma_{\rm mm}}{r}\right)^{12} - \left(\frac{\sigma_{\rm mm}}{r}\right)^{6}\right] + \varepsilon_{\rm mm}, \tag{S2}$$

where $\varepsilon_{\rm mm}$ and $\sigma_{\rm mm}$ are the energy and length scales associated with the LJ potential and the subscript "mm" indicates the interactions between monomer beads. The first term of Equation (S2) extends to R_0, with the common choices of $k_b = 30\varepsilon_{\rm mm}/\sigma_{\rm mm}^2$ and $R_0 = 1.5\sigma_{\rm mm}$, and the second term has a cutoff at $2^{1/6}\sigma_{\rm mm}$.

Additional intramolecular potentials include a bending potential in relation to chain rigidity [47],

$$U_{\mathrm{bend}}(\theta) = -A \sin^2(B\theta), \tag{S3}$$

where θ is the bond angle between two consecutive bonds. Based on our previous work [48], the parameter associated with the rest angle is fixed at $B = 1.5$ and the parameter A is varied to tune chain rigidity.

Finally, charged beads interact via a long-range Coulomb potential,

$$U_{\mathrm{Coul}}(r) = \frac{q_i q_j}{4\pi\varepsilon_0\varepsilon_r r}, \tag{S4}$$

where q_i and q_j are the charges of the interacting ion pairs i and j, which are set to be $+1e$ and $-1e$ for cationic and anionic beads with e being the elementary charge, ε_0 is the vacuum permittivity, and ε_r is the dielectric constant of the background medium.

S2.2. Details about specific simulated systems

The KA system [49, 50] is composed of two particle species A and B with equal mass m that interact through the LJ potential [Equation (S1)]. The system contains a total number of $N = 4000$ particles and has the composition of $N_A/N_B = 4:1$. The interaction and size parameters are $\varepsilon_{AB}/\varepsilon_{AA} = 1.5$, $\varepsilon_{BB}/\varepsilon_{AA} = 0.5$, $\sigma_{AB}/\sigma_{AA} = 0.8$, and $\sigma_{BB}/\sigma_{AA} = 0.88$. Our results are obtained over a range of fixed number densities from $\rho = N/V = 1.2\sigma^{-3}$ to $2\sigma^{-3}$ with V being the volume and along an isobaric path over a wide range of pressures P from $0\varepsilon/\sigma^3$ to $400\varepsilon/\sigma^3$. For more details, the reader is referred to Refs. 10 and 51.

The linear polymer melts are modeled using chains of variable length ($3 \leq M \leq 30$) and chain rigidity (A) and at different P [19]. The LJ potential [Equation (S1)] is used to model the interactions between any two nonbonded beads. Neighboring beads along a chain are bonded to each other through the FENE potential [Equation (S2)]. Moreover, chain rigidity can be controlled by applying an angular potential to two consecutive bonds [Equation (S3)]. For more details, the reader is referred to Ref. 19.

The star polymers have f arms ($f = 2$ to 20) with M_a beads per arm and the total mass is fixed at $M = fM_a + 1 = 61$ [52]. The core bead connects f arms. The interactions between nonbonded and bonded beads are described by the LJ and FENE potentials [Equations (S1)

and (S2)]. Our system consists of $N = 200$ star polymer chains and the pressure is fixed at $P = 0.0\varepsilon/\sigma^{-3}$. For more details, the reader is referred to Ref. 52.

The knotted ring polymers with $M = 36$ beads are initialized with polynomial knots of minimal crossing numbers varying from $m_c = 0$ to 7 [53]. Knot configurations follow the Alexander-Briggs classification. The system contains $N = 9000$ beads interacting with the LJ and FENE potentials [Equations (S1) and (S2)] for nonbonded and bonded interactions. For more details, the reader is referred to Ref. 53.

The charged polymer melts are composed of neutral chains with every other bead being attached by an anionic pendant ion [54, 55]. The anions are neutralized with an equal number of cationic free counterions. Each chain has 20 neutral backbone beads and 10 anionic beads. The system includes 200 chains and 2000 counterions, and hence, a total number of $N = 8000$ beads. Except for the LJ and FENE potentials [Equations (S1) and (S2)] describing nonbonded and bonded interactions, electrostatic interactions are treated via the Coulomb potential [Equation (S4)]. The strength of charge interactions can be characterized in terms of the Bjerrum length [56–59], $l_{\mathrm{B}} \equiv e^2/(4\pi\varepsilon_0\varepsilon_r k_{\mathrm{B}}T)$. To transform the model parameters into real units, we choose $\sigma = 1$ nm at room temperature as our unit of length. For more details, the reader is referred to Refs. 54 and 55.

The polymer-additive systems consist of 600 chains of length 10 at $P = 0.0\varepsilon/\sigma^{-3}$ [60, 61]. All nonbonded interactions are described by the LJ potential [Equation (S1)] with $\sigma_{\mathrm{mm}} = \varepsilon_{\mathrm{mm}} = 1.0$ in LJ units, and all bonded beads are interacted via the FENE potential [Equation (S2)]. The subscripts "m" and "a" denote monomer beads and additive particles, respectively. Additives are spherical particles with adjustable sizes $\sigma_{\mathrm{aa}}/\sigma$ varying from 0.6 to 1.8, where $\sigma_{\mathrm{ma}} = (\sigma_{\mathrm{mm}} + \sigma_{\mathrm{aa}})/2$. Interactions are chosen to prevent phase separation, $\varepsilon_{\mathrm{aa}} = 1.0$ and $\varepsilon_{\mathrm{ma}} = 1.5$. The additive volume fraction $v = N_{\mathrm{a}}\sigma_{\mathrm{aa}}^3/(N_{\mathrm{a}}\sigma_{\mathrm{aa}}^3 + N_{\mathrm{m}}\sigma_{\mathrm{mm}}^3) = 0.1$ is maintained by varying N_{a} from 114 to 3086 when σ_{aa} is altered. For more details, the reader is referred to Refs. 60 and 61.

All quantities and results are described in standard reduced LJ units. Specifically, length, time, temperature, and pressure are, respectively, given in units of σ, τ_{ref}, $\varepsilon/k_{\mathrm{B}}$, and ε/σ^3, where $\tau_{\mathrm{ref}} = \sqrt{m_b\sigma^2/\varepsilon}$ with m_b being the bead mass. Note that the units correspond to the LJ parameters of the type A particles for the KA system and of the monomer beads for the polymer-additive system. For the KA system, the reduced time τ_{ref}, reduced pressure ϵ/σ^3, reduced length σ, and reduced temperature $\varepsilon/k_{\mathrm{B}}$ are approximately 2 ps, 42 MPa,

0.3405×10^{-9} m, and 119.8 K, respectively [10].

S2.3. Simulation details

All the simulations are performed in three dimensions under periodic boundary conditions using the Large-scale Atomic/Molecular Massively Parallel Simulator (LAMMPS) molecular dynamics package [62, 63], where the pressure P and temperature T are maintained by a Nosé-Hoover barostat and thermostat [64, 65] and a time step of $\Delta t = 0.005\tau_{\mathrm{ref}}$ is used to integrate the equations of motion, except for the KA system at high ρ and P where we use $\Delta t = 0.002\tau_{\mathrm{ref}}$ [10, 51].

We initially prepare an equilibrated system at a relatively high temperature T_h, which is then quenched to the target temperatures at a cooling rate of $10^{-4}\varepsilon/(k_{\mathrm{B}}\tau_{\mathrm{ref}})$. For the KA system under constant V conditions, our simulations are performed over a range of fixed number densities in the canonical (NVT) ensemble. For other systems under constant P conditions, our results are obtained along an isobaric path, as described below. To obtain $\rho = N/V$ as a function of T and study glass formation upon cooling, the equilibrated melt is subsequently subjected to a cooling or heating process at a given P in the NPT ensemble by changing the temperature from T_h to lower or higher values at a rate of $10^{-4}\varepsilon/(k_{\mathrm{B}}\tau_{\mathrm{ref}})$. As expected, the T dependence of ρ (or V) exhibits a change in slope at some T defining the glass transition temperature T_{g}, which indicates that the system falls out of equilibrium at low T. For each T studied, properties are calculated in the NVT ensemble after the initial configuration, taken from the cooling or heating process, is further equilibrated for a period typically over 10 or 100 times longer than the segmental structural relaxation time τ_α from the self-intermediate scattering function.

S3. BASIC REFERENCE PROPERTIES

Based on previous works, the time scale of "immobile" particles is quantified by the segmental structural relaxation time τ_α, while the time scale of "mobile" particles is quantified by the peak time t^* of the non-Gaussian parameter [14–16]. Specifically, τ_α is determined

based on the self-intermediate scattering function,

$$F_s(q,t) = \frac{1}{N}\left\langle \sum_{j=1}^{N} \exp\{-\boldsymbol{i}\mathbf{q}\cdot[\mathbf{r}_j(t)-\mathbf{r}_j(0)]\} \right\rangle, \tag{S5}$$

where i is the imaginary unit, $q = |\boldsymbol{q}|$ is the wave number, $\boldsymbol{r}_j$ is the position of particle j, t is the time, and the angular brackets $\langle ... \rangle$ denote the usual thermal average. The wave number is chosen to be $q = 7.0\sigma^{-1}$ for the polymeric systems and $q = 7.2\sigma^{-1}$ for the KA system, which are close to the first peak positions of the static structure factor $S(q)$. τ_α is defined by the time at which $F_s(q,t)$ decays to 0.2.

To estimate the fast β-relaxation time $\tau_{\beta,f}$, we fit $F_s(q,t)$ to a two-step relaxation function [52, 66, 67],

$$F_s(q,t) = (1-A_\alpha)\exp\left[-(t/\tau_{\beta,f})^{\beta_f}\right] + A_\alpha \exp\left[-(t/\tau_{\alpha,\mathrm{fit}})^{\beta}\right], \tag{S6}$$

where A_α, β_f, and β are fitting parameters. As in previous studies of polymeric and non-polymeric GF liquids, the T dependence of $\tau_{\beta,f}$ is relatively weak.

The non-Gaussian parameter is defined as,

$$\alpha_2(t) = \frac{3\langle r^4(t)\rangle}{5\langle r^2(t)\rangle^2} - 1, \tag{S7}$$

where $\langle r^2(t)\rangle$ ($\langle r^4(t)\rangle$) is the mean squared (quartered) distance of particles,

$$\left\langle r^2(t)\right\rangle = \frac{1}{N}\left\langle \sum_{j=1}^{N} |\boldsymbol{r}_j(t) - \boldsymbol{r}_j(0)|^2 \right\rangle. \tag{S8}$$

A primary peak a_2^* is generally exhibited in $\alpha_2(t)$ at the peak time t^*, which has been shown to correspond to the lifetime of "mobile" particles [14–16, 68].

The string-like cooperative motion is determined by the number of mobile particles that move in a collective exchange fashion. Following standard practice [16], the mobile particles are selected as the fraction $f_0 = 6.5\%$ of particles with the greatest displacement over any chosen interval. In particular, two mobile particles j and k are defined to be in the same string if the following proximity condition holds:

$$\min[|\mathbf{r}_j(t)-\mathbf{r}_k(0)|, |\mathbf{r}_k(t)-\mathbf{r}_j(0)|] < \delta, \tag{S9}$$

where δ is a displacement distance parameter that the present work chooses to be 0.55. The number-averaged string length $\langle s(t)\rangle$ is then calculated via the relation,

$$\langle s(t)\rangle = \frac{\langle \sum_{s=1}^{\infty} s\, C(s)\rangle}{\sum_{s=1}^{\infty} C(s)}, \tag{S10}$$

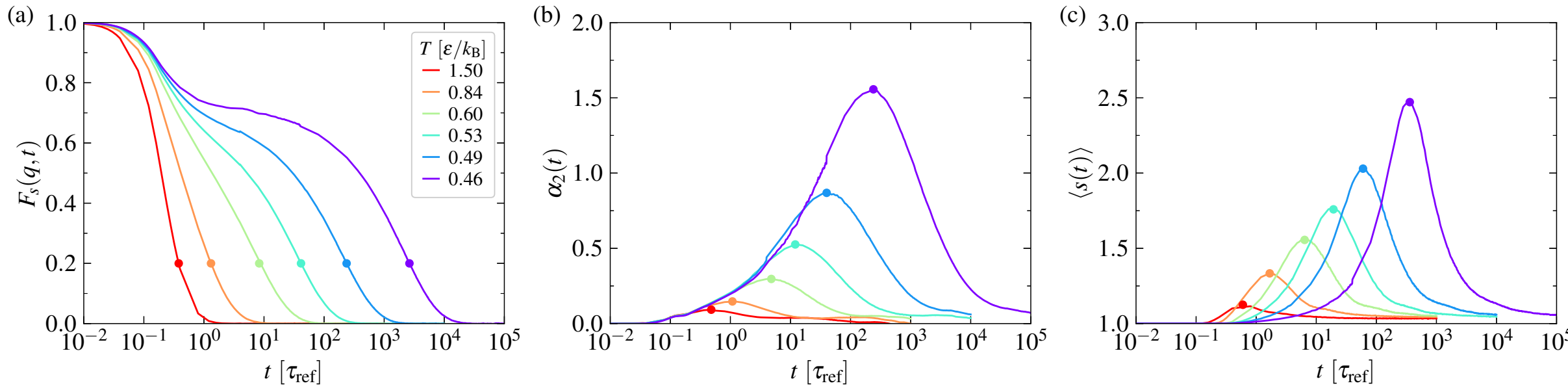

FIG. S1. Basic dynamic properties considered in the present work. Panels (a–c) show the self-intermediate scattering function $F_s(q,t)$, the non-Gaussian parameter $\alpha_2(t)$, and the number-averaged string length $\langle s(t) \rangle$ as a function of time t for a range of T for a polymer-additive system with $\sigma_{\rm aa} = 1.0\sigma$ and $\varepsilon_{\rm ma} = 1.5$, respectively. The circles indicate the structural relaxation times τ_α, the peak times t^* of $\alpha_2(t)$, and the peak times t_L of $\langle s(t) \rangle$.

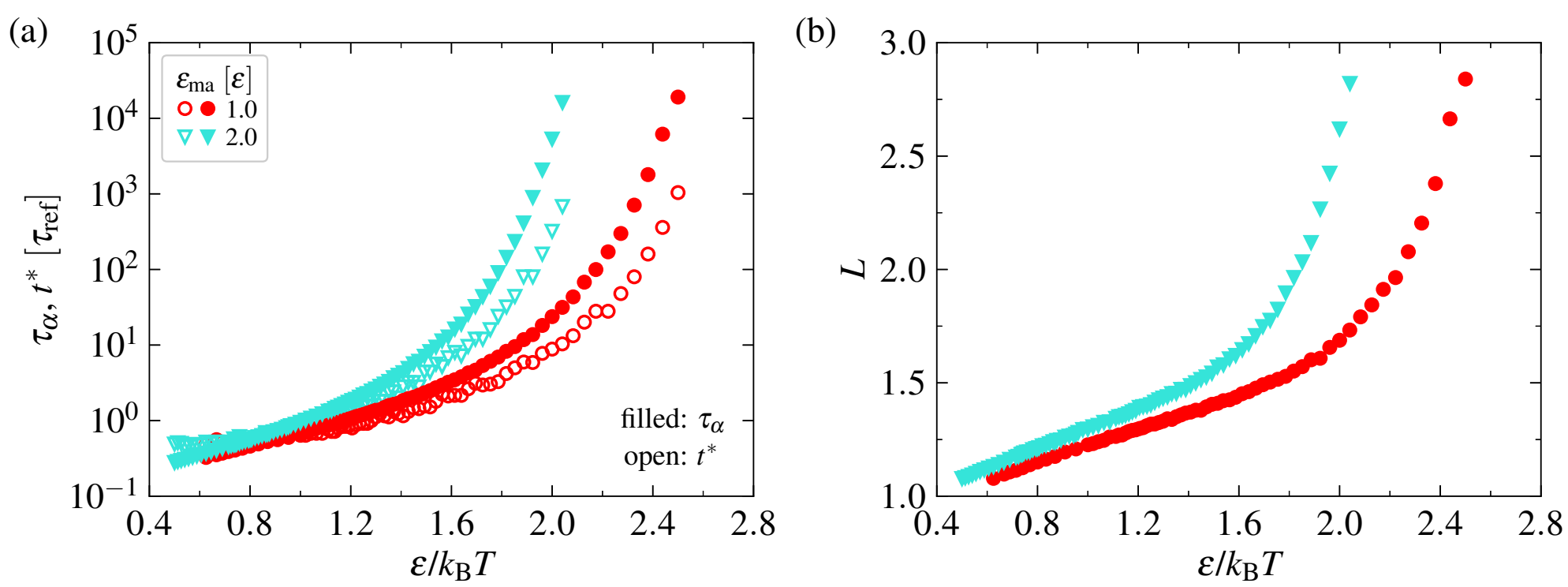

FIG. S2. Temperature dependence of basic properties. Panels (a) and (b) show τ_α and t^* of monomer beads and the average string length L for a polymer-additive system with varying interaction strength $\varepsilon_{\rm ma}$ between the polymer matrix and the additives.

where $C(s)$ is the number of strings of length s. The quantity $\langle s(t) \rangle$ exhibits a maximum value $L \equiv \langle s(t_L) \rangle$ at a characteristic time t_L, where L represents the characteristic string length that defines the spatial extent of string-like collective motion.

As an example, Figure S1 exhibits $F_s(q,t)$, $\alpha_2(t)$, and $\langle s(t) \rangle$ as a function of t for a range of T in a polymer-additive system with $\upsilon = 0.1$, $\sigma_{\rm aa} = 1.0\sigma$, and $\varepsilon_{\rm ma} = 1.5\varepsilon$ at $P = 0.0\varepsilon/\sigma^3$. The dynamics of the system slow down drastically upon cooling, which is typical of GF liquids. Figure S2 exhibits the T dependences of τ_α and t^* for monomers and additives, along with the T dependence of L. As can be seen, τ_α increases more rapidly than t^*, so that the persistence of the "immobile" particle clusters tends to become progressively longer

than "mobile" particle clusters [16, 17].

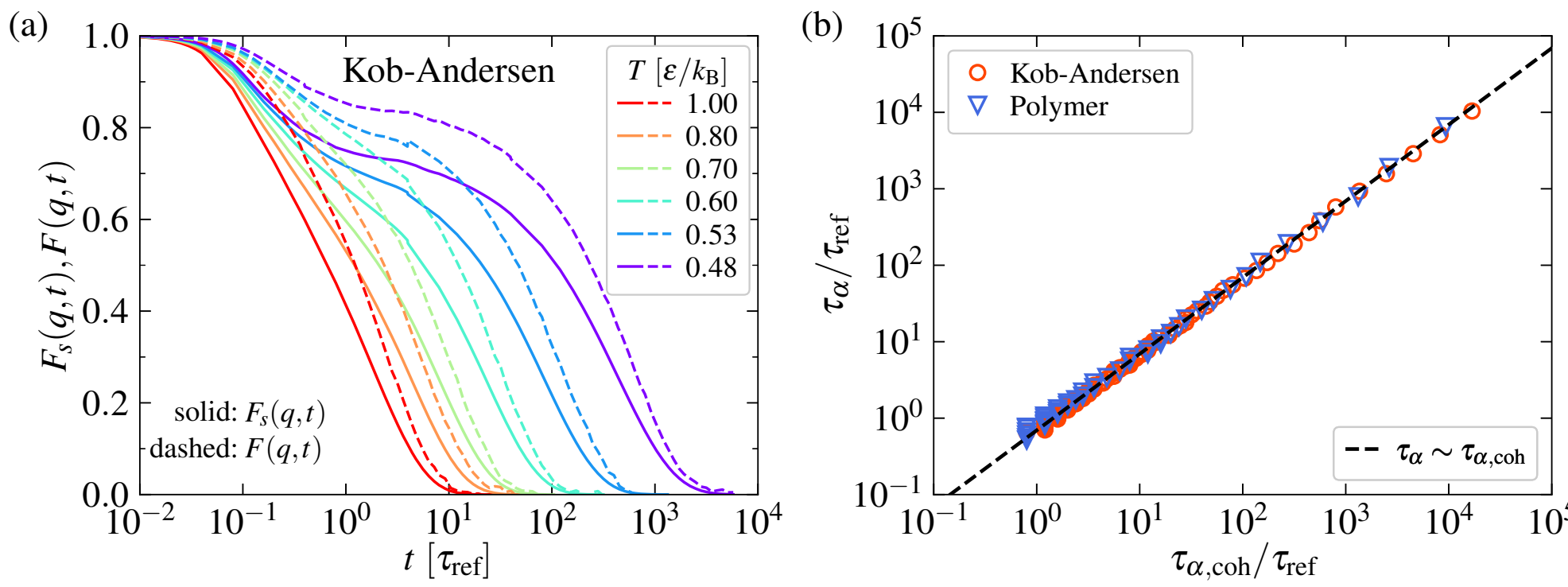

FIG. S3. Relationship between the coherent intermediate scattering function $F(q,t)$ and the self-intermediate scattering function $F_s(q,t)$. Panel (a) presents $F(q,t)$ and $F_s(q,t)$ as a function of t at varying T for the Kob-Andersen system. Panel (b) shows that the relaxation times $\tau_{\alpha,\rm coh}$ and τ_α are proportional to each other within numerical uncertainty in both the Kob-Andersen and polymer fluids at the interparticle distance.

Finally, we make a remark on the coherent intermediate scattering function $F(q,t)$. It is well established that the relaxation behavior defined via $F(q,t)$ is more intuitively suitable for characterizing structural relaxation and exhibits a strong correlation with the primary temperature dependence of shear viscosity. Specifically, $F(q,t)$ is defined by

$$F(q,t) = \frac{1}{NS(q)} \left\langle \sum_{j=1}^{N} \sum_{k=1}^{N} \exp\{-\boldsymbol{i}\mathbf{q} \cdot [\mathbf{r}_k(t) - \mathbf{r}_j(0)]\} \right\rangle. \tag{S11}$$

The wave number is chosen to be close to the first peak positions of the static structure factor $S(q)$,

$$S(q) = \frac{1}{N} \left\langle \sum_{j=1}^{N} \sum_{k=1}^{N} \exp[-i\mathbf{q} \cdot (\mathbf{r}_j - \mathbf{r}_k)] \right\rangle. \tag{S12}$$

Here, we compute $F(q,t)$ for the standard KA system and a representative polymer model having a chain length of $M = 10$ at $P = 0.0\varepsilon/\sigma^{-3}$. The relaxation time defined from $F(q,t)$, $\tau_{\alpha,\rm coh}$, is compared with the relaxation time τ_α obtained from $F_s(q,t)$. As shown in Fig. S3, $\tau_{\alpha,\rm coh}$ closely tracks τ_α at the interparticle distance, validating our use of τ_α as a surrogate for $\tau_{\alpha,\rm coh}$ and the shear structural relaxation time obtained from the shear stress relaxation function.

S4. DECOUPLING RELATION BETWEEN STRUCTURAL RELAXATION TIME AND MASS DIFFUSION COEFFICIENT

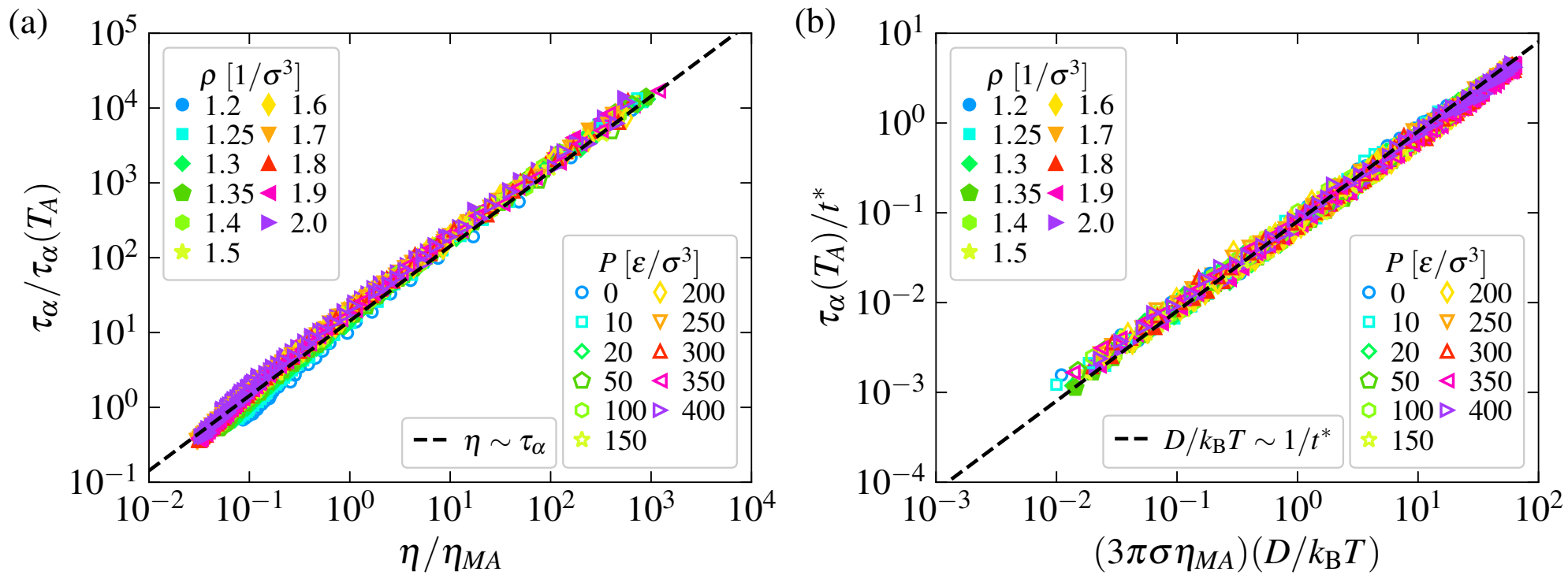

FIG. S4. Relation between the characteristic timescales and the mass and momentum diffusion coefficients in the Kob-Andersen system. Panel (a) shows the relation between the structural relaxation time τ_α and the viscosity η. The line indicates the relation, $\tau_\alpha/\tau_\alpha(T_A) \sim \eta/\eta_{MA}$. Here, η_{MA} is defined as $\eta_{MA} = G_\infty(T_A)\tau_\alpha(T_A)$, where G_∞ is the high-frequency shear modulus determined from the stress autocorrelation function $G(t)$, $G_\infty = G(t \to 0^+) = V/k_\mathrm{B}T\langle\sigma_{xy}(0^+)\sigma_{xy}(0)\rangle$. Panel (b) shows the relation between the self-diffusion coefficient D of the larger A particles divided by T and the peak time t^* of the non-Gaussian parameter. The line indicates the relation, $\tau_\alpha(T_A)/t^* \sim (3\pi\sigma\eta_{MA})(D/k_\mathrm{B}T)$.

In this section, we demonstrate the connection between the characteristic timescales (τ_α and t^*) in the KA system and the mass and momentum diffusion coefficients (D and η) [10], respectively. As shown in Fig. S4, τ_α varies nearly linearly with η, and t^* varies nearly linearly with the reciprocal of D/T over a wide range of temperatures under fixed density and pressure conditions. The constants in these plots are highly state dependent and are described in Ref. 10 and briefly in the figure caption. Note that some deviation from the straight lines in these figures exists in the "unstable" case of low or vanishing pressure.

S5. INFLUENCE OF ACTIVATION ENTROPY ON THE STRING MODEL FITS AND ITS GENERAL PHYSICAL SIGNIFICANCE

The central quantity in TST [69, 70] is the activation free energy, ΔG_o, which includes both enthalpic ΔH_o and entropic ΔS_o contributions. The entropic contribution is notori-

ously difficult to estimate theoretically in TST, so both the AG theory [71] and the GET [42] assume as a rough approximation that the entropic contribution is neglected, i.e., $\Delta S_o = 0$. This approximation has been frequently avoided in the string model, which complicates the analysis of the model, but also improves the comparison of the model to simulation observations. The estimation of ΔS_o in the particular case of polymer materials has been discussed for many years. A discussion of this quantity in the case of alkanes was given more recently by Jeong and Douglas [72] in the Arrhenius regime above T_A and was made by Eby [73] for a range of polymer materials in their solid state long ago. We also mention Ref. 74 as being representative of the application of TST to polymeric materials.

It should be appreciated that the activation free energy describes changes in the free energy of the system associated with the energetic cost of barrier crossing events required for molecular transport, so that the enthalpy and entropy of activation have no evident general connection to measured thermodynamic properties of the material, such as the observed enthalpy and entropy of the fluid. The TST treatment of simpler systems, such as crystalline materials and small clusters, gives insight into these energetic parameters [75–79], although these parameters must normally be determined in practice for most materials by numerical TST calculations or directly from measurements. Estimation of ΔH_o is normally straightforward from the determination of the slope of the Arrhenius plot describing the temperature dependence of the relaxation time, diffusion, or other kinetic properties. This energetic parameter, reflecting both the stable modes associated with free energy minima, and unstable normal modes associated with transition state events on the free energy surface, has often been found to correlate with the heat of vaporization and other measures of the molecular cohesive energy [72]. However, the entropy of activation ΔS_o is a much more difficult property to estimate and correlate with thermodynamic properties, and its estimation has been one of the persistent problems in TST since its original formulation. At a practical level, it is helpful that ΔH_o and ΔS_o are often found to vary in a concerted fashion, a phenomenon termed the entropy-enthalpy compensation (EEC) [72], but the absence of a general analytic theory predicting ΔS_o and this often observed, but not universal, compensation effect has also persisted as a fundamental problem in understanding the dynamics of condensed materials. Gelin *et al.* [79] recently provided some insight into TST calculations of model condensed liquids.

Within the framework of the string model, the activation free energy appears in the

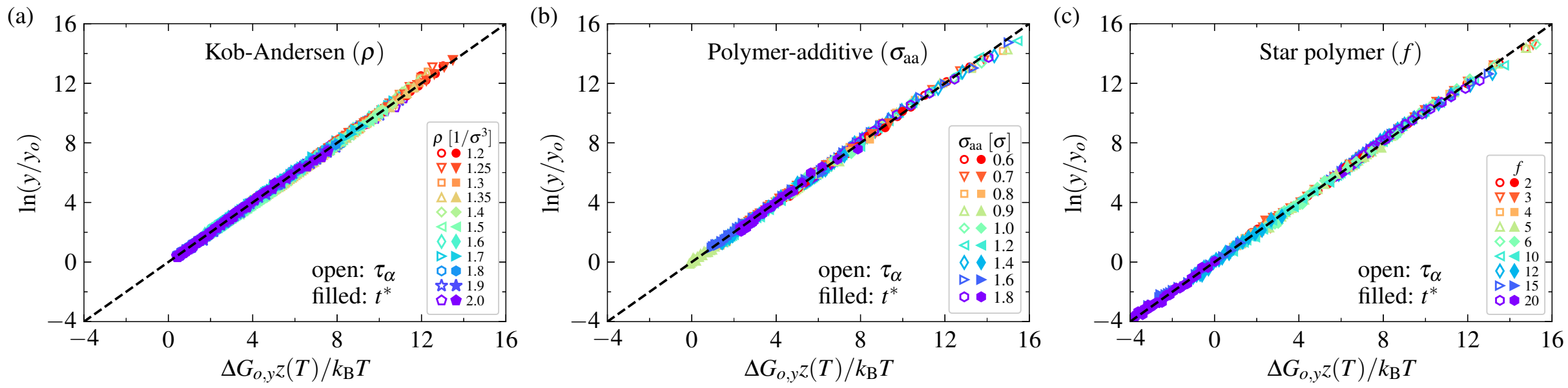

FIG. S5. String model fits for the structural relaxation time τ_α and the peak time t^* of the non-Gaussian parameter for various systems when the activation entropy is included. Panels (a–c) show the results for the KA system with varying density ρ, a polymer-additive system with varying additive size σ_{aa}, and a star polymer melt with varying arm number f, respectively. y refers to either τ_α or t^*. Lines indicate the string model relations.

expression,

$$y = y_o \exp\left[\frac{\Delta G_{o,y}}{k_{\text{B}}T} z(T)\right], \tag{S13}$$

where y refers to a specific property, which is either τ_α or t^* in the present study. The prefactors y_o can be eliminated by using the simulation data at the onset temperature T_A. Expanding the enthalpic and entropic contributions to $\Delta G_{o,y}$ leads to the following expression,

$$y = y(T_A) \exp\left[\frac{\Delta H_{o,y} - T\Delta S_{o,y}}{k_{\text{B}}T} z(T) - \frac{\Delta H_{o,y} - T_A \Delta S_{o,y}}{k_{\text{B}}T_A}\right], \tag{S14}$$

where the term $\Delta H_{o,y}$ is obtained by fitting the Arrhenius relationship in the high temperature regime. This treatment leaves $\Delta S_{o,y}$ as the sole free parameter in the equation. Since $\Delta G_{o,y} = \Delta H_{o,y} - T\Delta S_{o,y}$, the free energy calculated from this procedure is a temperature-dependent quantity, which makes it difficult to readily quantify the decoupling relation in terms of $\Delta G_{o,y}$.

To simplify the analysis of the string model, we take the activation free energies for mass diffusion associated with t^* and momentum diffusion [80–83] associated with τ_α to be constants in the high temperature regime above T_A. This simplifying assumption is made based on "mathematical expediency" in the AG model [71]. As an example, Fig. S5 shows the test of the string model when the activation entropy $\Delta S_{o,y}$ is included for the KA system with varying density ρ, a polymer-additive system with varying additive size σ_{aa}, and a star polymer melt with varying arm number f. This result indicates that our assumption of

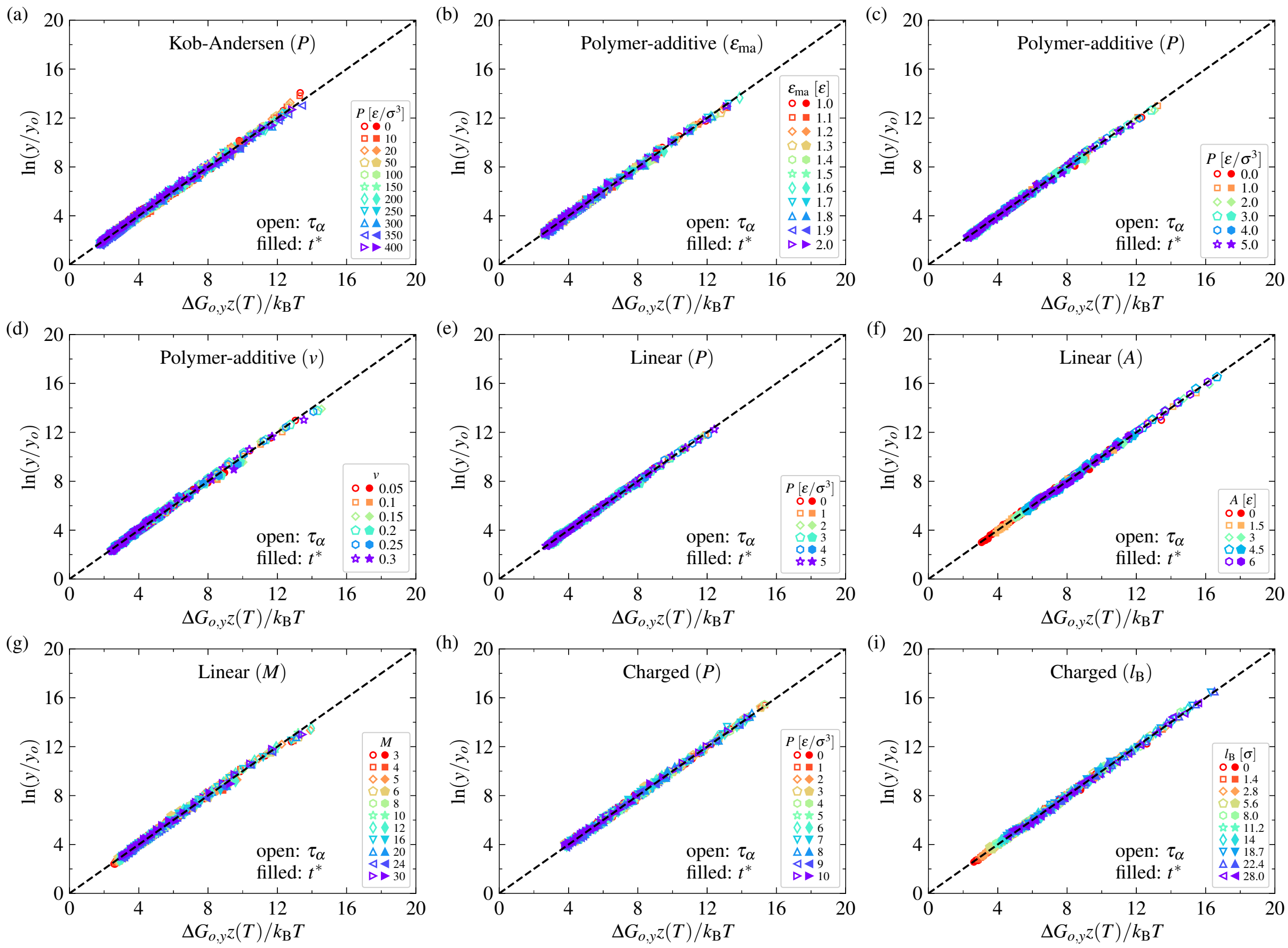

FIG. S6. Summary of the string model fits for various systems when the activation entropy is neglected. Panels (a–i) correspond to the results for the KA system with varying P, a polymer-additive system with varying $\varepsilon_{\rm ma}$, P, and volume fraction v, a linear polymer melt [19] with varying P, chain rigidity A, and molecular mass M, a charged polymer melt with varying P [55] and "Bjerrum length" $l_{\rm B}$ [54], respectively. y refers to either τ_α or t^*. Lines indicate the string model relations.

taking $\Delta G_{o,y}$ as a constant does not significantly degrade the quality of the fits to the string model.

For completeness, we summarize the string model fits for τ_α and t^* for other systems that are not presented in the main text. As shown in Fig. S6, the string model can excellently describe both τ_α and t^* for all the systems that we simulate when the activation entropy is neglected.

S6. SUMMARY OF THE DECOUPLING EXPONENT FOR VARIOUS SYSTEMS

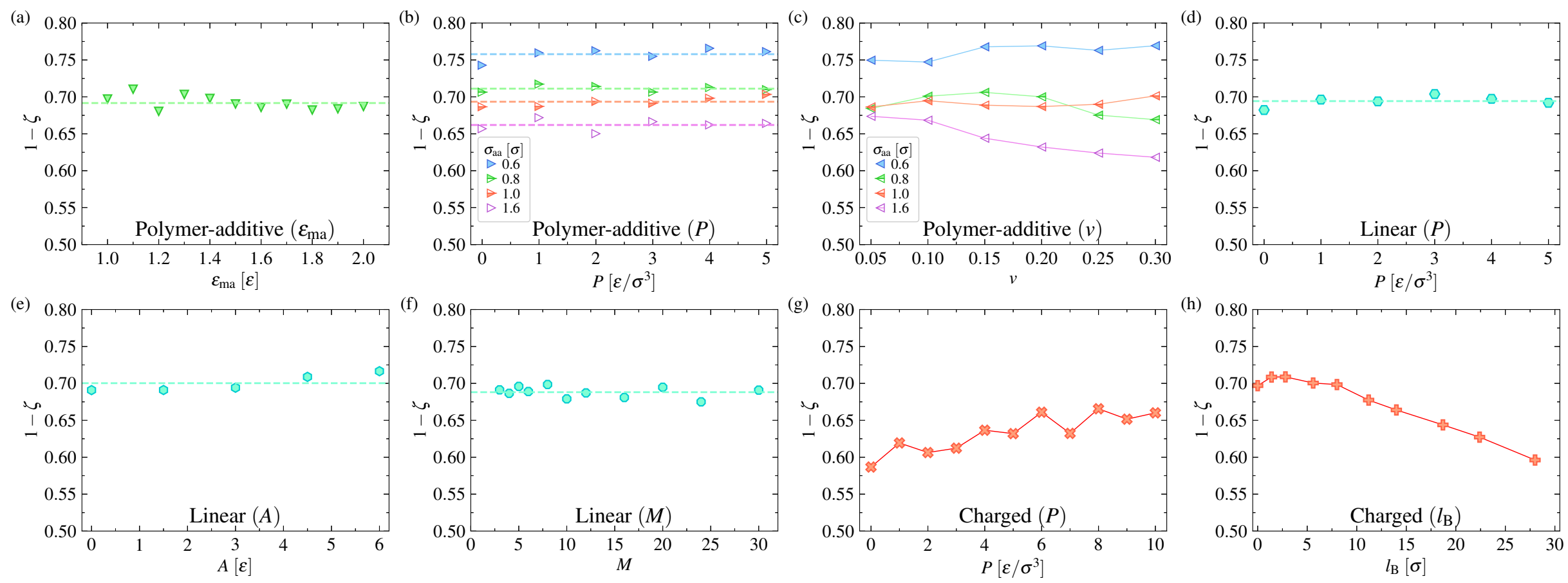

FIG. S7. Summary of the decoupling exponent $1-\zeta$ for various systems. Panels (a–h) correspond to the results for a polymer-additive system with varying $\varepsilon_{\mathrm{ma}}$, P, and volume fraction v, a linear polymer melt with varying P, chain rigidity A, and molecular mass M, a charged polymer melt with varying P and Bjerrum length l_{B}, respectively. The specification of the parameter values used in our simulations is given in the main text. Except when the parameter A is varied, all polymers considered remain fully flexible, i.e., $A=0$. Dashed lines are a guide to the eye.

This section summarizes the decoupling exponent $1-\zeta$ as functions of molecular and thermodynamic parameters determined from the direct analysis of the power-law relation between τ_α and t^*. These data are shown in Fig. S7 for various systems. We note that ζ has been observed by dielectric spectroscopy to vary with M when the chains are oligomeric, but not long enough to have a random coiled structure of polymers [29]. Our coarse-grained model of polymers is limited to modelling polymers and cannot address changes in ζ occurring in oligomers. A detailed discussion of these results is left for future work.

---

[1] Plazek DJ. Temperature dependence of the viscoelastic behavior of polystyrene. *J. Phys. Chem.* 1965; **69**: 3480–3487.

[2] Ding Y and Sokolov AP. Breakdown of time-temperature superposition principle and universality of chain dynamics in polymers. *Macromolecules* 2006; **39**: 3322–3326.

[3] Sokolov A and Hayashi Y. Breakdown of time-temperature superposition: From experiment to the coupling model and beyond. *J. Non-Cryst. Solids* 2007; **353**: 3838–3844.

[4] Xu X, Douglas JF and Xu WS. Generalized entropy theory investigation of the relatively high segmental fragility of many glass-forming polymers. *Soft Matter* 2025; **21**: 2664–2685.

[5] Sokolov AP, Novikov VN and Ding Y. Why many polymers are so fragile. *J. Phys. Condens. Matter* 2007; **19**: 205116.

[6] Dalle-Ferrier C, Niss K, Sokolov AP *et al.* The role of chain length in nonergodicity factor and fragility of polymers. *Macromolecules* 2010; **43**: 8977–8984.

[7] Dalle-Ferrier C, Kisliuk A, Hong L *et al.* Why many polymers are so fragile: A new perspective. *J. Chem. Phys.* 2016; **145**: 154901.

[8] Agapov AL, Novikov VN, Hong T *et al.* Surprising temperature scaling of viscoelastic properties in polymers. *Macromolecules* 2018; **51**: 4874–4881.

[9] Urakawa O, Swallen SF, Ediger MD *et al.* Self-diffusion and viscosity of low molecular weight polystyrene over a wide temperature range. *Macromolecules* 2004; **37**: 1558–1564.

[10] Yuan QL, Xu X, Douglas JF *et al.* Influence of density and pressure on glass formation in the Kob-Andersen model. *J. Phys. Chem. B* 2024; **128**: 9889–9904.

[11] Johari GP and Goldstein M. Viscous liquids and the glass transition. II. Secondary relaxations in glasses of rigid molecules. *J. Chem. Phys.* 1970; **53**: 2372–2388.

[12] Johari GP and Goldstein M. Viscous liquids and the glass transition. III. Secondary relaxations in aliphatic alcohols and other nonrigid molecules. *J. Chem. Phys.* 1971; **55**: 4245–4252.

[13] Johari GP. Intrinsic mobility of molecular glasses. *J. Chem. Phys.* 1973; **58**: 1766–1770.

[14] Zhang H, Wang X, Yu HB *et al.* Fast dynamics in a model metallic glass-forming material. *J. Chem. Phys.* 2021; **154**: 084505.

[15] Zhang H, Wang X, Yu HB *et al.* Dynamic heterogeneity, cooperative motion, and Johari–Goldstein β-relaxation in a metallic glass-forming material exhibiting a fragile-to-strong transition. *Eur. Phys. J. E* 2021; **44**: 56.

[16] Starr FW, Douglas JF and Sastry S. The relationship of dynamical heterogeneity to the Adam-Gibbs and random first-order transition theories of glass formation. *J. Chem. Phys.* 2013; **138**: 12A541.

[17] Zhang H, Zhong C, Douglas JF *et al.* Role of string-like collective atomic motion on diffusion and structural relaxation in glass forming Cu-Zr alloys. *J. Chem. Phys.* 2015; **142**: 164506.

[18] Xu X, Douglas JF and Xu WS. Thermodynamic–dynamic interrelations in glass-forming polymer fluids. *Macromolecules* 2022; **55**: 8699–8722.

[19] Xu X, Douglas JF and Xu WS. Parallel emergence of rigidity and collective motion in a family of simulated glass-forming polymer fluids. *Macromolecules* 2023; **56**: 4929–4951.

[20] Massa CA, Puosi F and Leporini D. Fractional coupling of primary and Johari–Goldstein relaxations in a model polymer. *Polymers* 2022; **14**: 5560.

[21] Douglas JF and Xu WS. Equation of state and entropy theory approach to thermodynamic scaling in polymeric glass-forming liquids. *Macromolecules* 2021; **54**: 3247–3269.

[22] Shahin Thayyil M, Ngai K, Prevosto D *et al.* Revealing the rich dynamics of glass-forming systems by modification of composition and change of thermodynamic conditions. *J. Non-Cryst. Solids* 2015; **407**: 98–105.

[23] Kessairi K, Capaccioli S, Prevosto D *et al.* Interdependence of primary and Johari-Goldstein secondary relaxations in glass-forming systems. *J. Phys. Chem. B* 2008; **112**: 4470–4473.

[24] Xu WS, Douglas JF and Freed KF. Influence of pressure on glass formation in a simulated polymer melt. *Macromolecules* 2017; **50**: 2585–2598.

[25] Masser KA, Long TR, Yu JH *et al.* The temperature-dependent ballistic performance and the ductile-to-brittle transition in polymer networks. *J. Polym. Sci., Part B: Polym. Phys.* 2019; **57**: 511–523.

[26] Ning R, Xu B, Wang S *et al.* Translational and rotational rearrangement regions governing β relaxations in polymer glasses. *J. Chem. Phys.* 2026; **164**: 064504.

[27] Fujimori H and Oguni M. Construction of an adiabatic calorimeter at low temperatures and glass transition of crystalline 2-bromothiophene. *J. Phys. Chem. Solids* 1993; **54**: 271–280.

[28] Kissi EO, Grohganz H, Löbmann K *et al.* Glass-transition temperature of the β-relaxation as the major predictive parameter for recrystallization of neat amorphous drugs. *J. Phys. Chem. B* 2018; **122**: 2803–2808.

[29] Mattsson J, Bergman R, Jacobsson P *et al.* Influence of chain length on the $\alpha-\beta$ bifurcation in oligomeric glass formers. *Phys. Rev. Lett.* 2005; **94**: 165701.

[30] Ngai KL, Capaccioli S, Prevosto D *et al.* Coupling of caged molecule dynamics to JG β-relaxation II: Polymers. *J Phys. Chem. B* 2015; **119**: 12502–12518.

[31] O'Connell PA and McKenna GB. Arrhenius-type temperature dependence of the segmental relaxation below tg. *J. Phys. Chem.* 1999; **110**: 11054–11060.

[32] Casalini R and Roland CM. Aging of the secondary relaxation to probe structural relaxation in the glassy state. *Phys. Rev. Lett.* 2009; **102**: 035701.

[33] Novikov VN and Sokolov AP. Qualitative change in structural dynamics of some glass-forming systems. *Phys. Rev. E* 2015; **92**: 062304.

[34] Reynolds M, Baker DL, Olmsted PD *et al.* Chain-length-dependent correlated molecular motion in polymers. *Phys. Rev. Lett.* 2025; **135**: 218101.

[35] Doremus RH. Viscosity of silica. *J. Appl. Phys.* 2002; **92**: 7619–7629.

[36] Ojovan MI, Travis KP and Hand RJ. Thermodynamic parameters of bonds in glassy materials from viscosity–temperature relationships. *J. Phys. Cond. Matter* 2007; **19**: 415107.

[37] Cerveny S, Alegría A and Colmenero J. Universal features of water dynamics in solutions of hydrophilic polymers, biopolymers, and small glass-forming materials. *Phys. Rev. E* 2008; **77**: 031803.

[38] Pazmiño Betancourt BA, Douglas JF and Starr FW. String model for the dynamics of glass-forming liquids. *J. Chem. Phys.* 2014; **140**: 204509.

[39] Xu WS, Douglas JF and Freed KF. Generalized entropy theory of glass-formation in fully flexible polymer melts. *J. Chem. Phys.* 2016; **145**: 234509.

[40] Zhao J, Simon SL and McKenna GB. Using 20-million-year-old amber to test the super-Arrhenius behaviour of glass-forming systems. *Nat. Commun.* 2013; **4**: 1783.

[41] Kong D, Meng Y and McKenna GB. Searching for the ideal glass transition: Going to yotta seconds and beyond. *J. Non-Cryst. Solids* 2023; **606**: 122186.

[42] Xu WS, Douglas JF and Sun ZY. Polymer glass formation: Role of activation free energy, configurational entropy, and collective motion. *Macromolecules* 2021; **54**: 3001–3033.

[43] Yu HB, Wang WH, Bai HY *et al.* The β-relaxation in metallic glasses. *Natl. Sci. Rev.* 2014; **1**: 429–461.

[44] Yu HB, Richert R and Samwer K. Structural rearrangements governing Johari-Goldstein relaxations in metallic glasses. *Sci. Adv.* 2017; **3**: e1701577.

[45] Kremer K and Grest GS. Dynamics of entangled linear polymer melts: A molecular-dynamics simulation. *J. Chem. Phys.* 1990; **92**: 5057–5086.

[46] Grest GS and Kremer K. Molecular dynamics simulation for polymers in the presence of a heat bath. *Phys. Rev. A* 1986; **33**: 3628–3631.

[47] Hsu HP and Kremer K. A coarse-grained polymer model for studying the glass transition. *J.*

Chem. Phys. 2019; **150**: 091101.

[48] Xu WS, Douglas JF and Xu X. Molecular dynamics study of glass formation in polymer melts with varying chain stiffness. *Macromolecules* 2020; **53**: 4796–4809.

[49] Kob W and Andersen HC. Scaling behavior in the β-relaxation regime of a supercooled lennard-jones mixture. *Phys. Rev. Lett.* 1994; **73**: 1376–1379.

[50] Kob W and Andersen HC. Testing mode-coupling theory for a supercooled binary Lennard-Jones mixture I: The van hove correlation function. *Phys. Rev. E* 1995; **51**: 4626–4641.

[51] Yuan QL, Xu X, Douglas JF *et al.* Understanding relaxation in the Kob-Andersen liquid based on entropy, string, shoving, localization, and parabolic models. *J. Phys. Chem. B* 2024; **128**: 10999–11021.

[52] Yang Z, Xu X, Douglas JF *et al.* Confinement effect of inter-arm interactions on glass formation in star polymer melts. *J. Chem. Phys.* 2024; **160**: 044503.

[53] Dong YT, Song XY, Xu X *et al.* Glass formation in ring polymer melts having variable knot complexity and molecular mass. *Macromolecules* 2024; **57**: 6875–6896.

[54] Yang Z, Xu X and Xu WS. Influence of ionic interaction strength on glass formation of an ion-containing polymer melt. *Macromolecules* 2021; **54**: 9587–9601.

[55] Yang Z, Xu X, Douglas JF *et al.* Molecular dynamics investigation of the pressure dependence of glass formation in a charged polymer melt. *Macromolecules* 2023; **56**: 4049–4064.

[56] Hall LM, Stevens MJ and Frischknecht AL. Effect of polymer architecture and ionic aggregation on the scattering peak in model ionomers. *Phys. Rev. Lett.* 2011; **106**: 127801.

[57] Hall LM, Seitz ME, Winey KI *et al.* Ionic aggregate structure in ionomer melts: Effect of molecular architecture on aggregates and the ionomer peak. *J. Am. Chem. Soc.* 2012; **134**: 574–587.

[58] Hall LM, Stevens MJ and Frischknecht AL. Dynamics of model ionomer melts of various architectures. *Macromolecules* 2012; **45**: 8097–8108.

[59] Li W and Olvera de la Cruz M. Glass transition of ion-containing polymer melts in bulk and thin films. *Soft Matter* 2021; **17**: 8420–8433.

[60] McKenzie-Smith TQ, Douglas JF and Starr FW. Explaining the sensitivity of polymer segmental relaxation to additive size based on the localization model. *Phys. Rev. Lett.* 2021; **127**: 277802.

[61] McKenzie-Smith T, Douglas JF and Starr FW. Relating dynamic free volume to cooperative

relaxation in a glass-forming polymer composite. *J. Chem. Phys.* 2022; **157**: 131101.

[62] Plimpton S. Fast parallel algorithms for short-range molecular dynamics. *J. Comput. Phys.* 1995; **117**: 1–19.

[63] LAMMPS web page: `http://lammps.sandia.gov`. The present work utilized the version released on 22/08/2018.

[64] Nosé S. A unified formulation of the constant temperature molecular dynamics methods. *J. Chem. Phys.* 1984; **81**: 511–519.

[65] Hoover WG. Canonical dynamics: Equilibrium phase-space distributions. *Phys. Rev. A* 1985; **31**: 1695–1697.

[66] Giuntoli A, Puosi F, Leporini D *et al.* Predictive relation for the α-relaxation time of a coarse-grained polymer melt under steady shear. *Sci. Adv.* 2020; **6**: eaaz0777.

[67] Pazmiño Betancourt BA, Starr FW and Douglas JF. String-like collective motion in the α- and β-relaxation of a coarse-grained polymer melt. *J. Chem. Phys.* 2018; **148**: 104508.

[68] Das P and Sastry S. Crossover in dynamics in the Kob-Andersen binary mixture glass-forming liquid. *J. Non-Cryst. Solids X* 2022; **14**: 100098.

[69] Glasstone S, Laidler KJ and Eyring H. *The Theory of Rate Processes: The Kinetics of Chemical Reactions, Viscosity, Diffusion and Electrochemical Phenomena*. International Chemical Series, (McGraw-Hill Book Company: Incorporated1941).

[70] Laidler KJ and King MC. Development of transition-state theory. *J. Phys. Chem.* 1983; **87**: 2657–2664.

[71] Adam G and Gibbs JH. On the temperature dependence of cooperative relaxation properties in glass-forming liquids. *J. Chem. Phys.* 1965; **43**: 139–146.

[72] Jeong C and Douglas JF. Mass dependence of the activation enthalpy and entropy of unentangled linear alkane chains. *J. Chem. Phys.* 2015; **143**: 144905.

[73] Eby RK. Empirical relations of thermodynamic "constants" to the activation parameters of polymer relaxations. *J. Chem. Phys.* 1962; **37**: 2785–2790.

[74] Brown A and Ashby M. Correlations for diffusion constants. *Acta Metall.* 1980; **28**: 1085–1101.

[75] Vineyard GH. Frequency factors and isotope effects in solid state rate processes. *J. Phys. Chem. Solids* 1957; **3**: 121–127.

[76] Rice SA. Dynamical theory of diffusion in crystals. *Phys. Rev.* 1958; **112**: 804–811.

[77] Kong LT and Lewis LJ. Transition state theory of the preexponential factors for self-diffusion

on cu, ag, and ni surfaces. *Phys. Rev. B* 2006; **74**: 073412.

[78] Maslov MM, Openov LA and Podlivaev AI. On the vineyard formula for the pre-exponential factor in the arrhenius law. *Phys. Solid State* 2014; **56**: 1239–1244.

[79] Gelin S, Champagne-Ruel A and Mousseau N. Enthalpy-entropy compensation of atomic diffusion originates from softening of low frequency phonons. *Nat. Commun.* 2020; **11**: 3977.

[80] Helfand E. Transport coefficients from dissipation in a canonical ensemble. *Phys. Rev.* 1960; **119**: 1–9.

[81] Meier K, Laesecke A and Kabelac S. Transport coefficients of the Lennard-Jones model fluid. I. Viscosity. *J. Chem. Phys.* 2004; **121**: 3671–3687.

[82] Viscardy S, Servantie J and Gaspard P. Transport and helfand moments in the Lennard-Jones fluid. I. Shear viscosity. *J. Chem. Phys.* 2007; **126**: 184512.

[83] Moustafa SG, Schultz AJ and Douglas JF. Efficient single-run implementation of generalized Einstein relation to compute transport coefficients: A binary-based time sampling. *J. Chem. Phys.* 2024; **160**: 024114.
